# Fundamentally different roles of neuronal TNF receptors in CNS pathology: TNFR1 and IKKβ promote microglial responses and tissue injury in demyelination while TNFR2 protects against excitotoxicity in mice

**DOI:** 10.1186/s12974-021-02200-4

**Published:** 2021-09-26

**Authors:** Irini Papazian, Eleni Tsoukala, Athena Boutou, Maria Karamita, Konstantinos Kambas, Lida Iliopoulou, Roman Fischer, Roland E. Kontermann, Maria C. Denis, George Kollias, Hans Lassmann, Lesley Probert

**Affiliations:** 1 Laboratory of Molecular Genetics, Department of Immunology, Hellenic Pasteur Institute, 127 Vasilissis Sophias Ave, 11521 Athens, Greece; 2 Institute of Cell Biology and Immunology, University of Stuttgart, Allmandring 31, 70569 Stuttgart, Germany; 3 Institute of Immunology, Biomedical Sciences Research Centre (BSRC) “Alexander Fleming”, Vari, 16672 Athens, Greece; 4 Department of Neuroimmunology, Center for Brain Research, Medical University of Vienna, Spitalgasse 4, A1090, Vienna, Austria

**Keywords:** Neuroinflammation, Immunotherapy, Multiple sclerosis, NF-κB, Autophagy, Neuroprotection, Neurodegeneration, Oligodendrocyte death, Glutamate excitotoxicity, TNFR2 agonist

## Abstract

**Background:**

During inflammatory demyelination, TNF receptor 1 (TNFR1) mediates detrimental proinflammatory effects of soluble TNF (solTNF), whereas TNFR2 mediates beneficial effects of transmembrane TNF (tmTNF) through oligodendroglia, microglia, and possibly other cell types. This model supports the use of selective inhibitors of solTNF/TNFR1 as anti-inflammatory drugs for central nervous system (CNS) diseases. A potential obstacle is the neuroprotective effect of solTNF pretreatment described in cultured neurons, but the relevance in vivo is unknown.

**Methods:**

To address this question, we generated mice with neuron-specific depletion of TNFR1, TNFR2, or inhibitor of NF-κB kinase subunit β (IKKβ), a main downstream mediator of TNFR signaling, and applied experimental models of inflammatory demyelination and acute and preconditioning glutamate excitotoxicity. We also investigated the molecular and cellular requirements of solTNF neuroprotection by generating astrocyte-neuron co-cultures with different combinations of wild-type (WT) and TNF and TNFR knockout cells and measuring N-methyl-d-aspartate (NMDA) excitotoxicity in vitro.

**Results:**

Neither neuronal TNFR1 nor TNFR2 protected mice during inflammatory demyelination. In fact, both neuronal TNFR1 and neuronal IKKβ promoted microglial responses and tissue injury, and TNFR1 was further required for oligodendrocyte loss and axonal damage in cuprizone-induced demyelination. In contrast, neuronal TNFR2 increased preconditioning protection in a kainic acid (KA) excitotoxicity model in mice and limited hippocampal neuron death. The protective effects of neuronal TNFR2 observed in vivo were further investigated in vitro. As previously described, pretreatment of astrocyte-neuron co-cultures with solTNF (and therefore TNFR1) protected them against NMDA excitotoxicity. However, protection was dependent on astrocyte, not neuronal TNFR1, on astrocyte tmTNF-neuronal TNFR2 interactions, and was reproduced by a TNFR2 agonist.

**Conclusions:**

These results demonstrate that neuronal TNF receptors perform fundamentally different roles in CNS pathology in vivo*,* with neuronal TNFR1 and IKKβ promoting microglial inflammation and neurotoxicity in demyelination, and neuronal TNFR2 mediating neuroprotection in excitotoxicity. They also reveal that previously described neuroprotective effects of solTNF against glutamate excitotoxicity in vitro are indirect and mediated via astrocyte tmTNF-neuron TNFR2 interactions. These results consolidate the concept that selective inhibition of solTNF/TNFR1 with maintenance of TNFR2 function would have combined anti-inflammatory and neuroprotective properties required for safe treatment of CNS diseases.

**Supplementary Information:**

The online version contains supplementary material available at 10.1186/s12974-021-02200-4.

## Introduction

Tumor necrosis factor (TNF) is a multifunctional cytokine system with diverse roles in the central nervous system (CNS), ranging from the regulation of physiological functions such as synaptic transmission and plasticity to the induction of inflammation, excitotoxicity, and oxidative tissue damage under disease conditions. TNF also has important beneficial functions in demyelinating diseases where it mediates the recruitment and differentiation of oligodendrocyte precursor cells (OPC) and remyelination [1, 2]. The relative contribution of beneficial over deleterious TNF functions to one human demyelinating disease, multiple sclerosis (MS), are clearly illustrated by clinical data, which show that non-selective TNF inhibitors exacerbate disease in MS patients [3] and induce de novo demyelination in patients treated for other diseases [4, 5]. Also, *TNFRSF1A* encodes a soluble form of TNF receptor 1 (TNFR1) that can block TNF and is a risk gene for MS [6, 7]. These data suggest that beneficial TNF functions in remyelination and suppression of inflammation outweigh deleterious ones and are important for CNS resistance to demyelination.

Understanding the mechanisms of TNF effect in the CNS is important for the refinement of TNF inhibitors as safe therapeutics for various inflammatory diseases including MS. The TNF system involves two ligands and two receptors [8]. Soluble TNF (solTNF) signals mainly through TNFR1 to mediate deleterious TNF effects [9, 10], including an inhibitory effect on remyelination [11]. Transmembrane TNF (tmTNF) signals mainly through TNF receptor 2 (TNFR2) and mediates beneficial effects, such as remyelination [12]. This information provides an important mechanistic framework upon which to base the design of novel selective TNF inhibitors for the treatment of CNS inflammatory diseases [13].

One potential obstacle for selective inhibition of solTNF/TNFR1 signaling as an anti-inflammatory therapy is its role in neuroprotection. Pretreatment of cultured neurons with solTNF (TNF preconditioning) protects them against a wide range of metabolic, excitotoxic, and oxidative death stimuli and promotes maintenance of calcium homeostasis [14, 15]. Whether these effects are exerted directly by TNF signaling in neurons, or indirectly by small numbers of glial cells in the cultures, and whether they are relevant in vivo remains unknown. Studies in conventional knockout mice showed worsening of CNS pathology in experimental stroke in TNFR1 knockout (TNFR1KO) mice [16], and in experimental autoimmune encephalomyelitis (EAE) and retinal ischemia in TNFR2KO mice [17–19], but the mechanisms underlying these protective effects are poorly understood. The availability of mutant mice carrying conditional TNFR1 and TNFR2 alleles now allows the cell specificity of TNF effects to be elucidated. Elegant studies recently showed that depletion of TNFR2 selectively in microglia [20] or oligodendroglia [21] led to early-onset or enhanced disease respectively in EAE, showing that glial cell-specific beneficial functions of TNFR2 are sufficient to protect the CNS during inflammatory demyelination.

While the effects of TNF in the CNS are predominantly mediated via non-neuronal cells, here we investigated the relevance of neuronal TNF receptors for CNS disease, especially their possible roles in neuroprotection. We generated mice with neuron-specific depletion of TNFR1, TNFR2, or inhibitor of NF-κB kinase subunit β (IKKβ), the main activating kinase for NF-κΒ in the canonical pathway and main downstream mediator of TNFR1 signaling [8, 22], and used them with models of inflammatory demyelination and glutamate excitotoxicity. We also used astrocyte-neuron co-cultures with different combinations of wildtype (WT), TNFKO, tmTNF knockin (tmTNFKI), and TNFRKO cells and glutamate excitotoxicity models in vitro, to verify and further define the molecular and cellular interactions underlying TNFR-mediated neuroprotection. We show that neuronal TNFR1 and TNFR2 have distinct inflammatory/neurotoxic and neuroprotective effects, in mouse models of CNS demyelination and excitotoxicity, respectively. Specifically, neuronal TNFR1 and IKKβ both advance the onset of inflammatory demyelination, neuronal TNFR1 is further required for axon damage and oligodendrocyte (OLG) loss during cuprizone-induced (CPZ) demyelination, and neuronal TNFR2 increases protection against glutamate excitotoxicity in vitro and in vivo. These results consolidate the concept that selective inhibition of solTNF/TNFR1 with maintenance of TNFR2 function would combine anti-inflammatory and neuroprotective properties required for the safe treatment of CNS disease.

## Materials and methods

### Animals

For in vitro experiments, male and female embryonic day (E) 14.5 embryos and postnatal day (P) 0 (first day after birth) mice were used for the isolation of cortical neurons and cortical astrocytes, respectively, from WT C57BL/6 (B6), *Tnf−/−* (TNFKO [23];), *tmTnfΔ/Δ* (tmTNFKI [24];, a gift of B. Ryffel, University of Orleans and CNRS, INEM, Molecular Immunology, Orleans, France), *Tnfrsf1a−/−* (TNFR1KO [25];, a gift of H. Bluethmann, Hoffmann-La Roche Ltd, Basel, Switzerland), and *Tnfrsf1b−/−* (TNFR2KO [26];) mice. For in vivo experiments, mice with conditional inactivation of TNFR1, TNFR2, or IKKβ selectively in CNS neurons were generated by Cre-*LoxP* recombination technology. The generation and initial characterization of TNFR1 conditional knockout mice has been described previously [27]. Briefly, mice in which exons 2–5 of the *Tnfrsf1a* gene sequence are flanked by *LoxP* sites (“floxed”) were generated using standard gene-targeting techniques (named here TNFR1ff mice). The generation and initial characterization of TNFR2 conditional knockout mice has been described previously [28]. Briefly, mice in which exon 2 of the *Tnfrsf1b* gene sequence is flanked by *LoxP* sites were generated using standard gene-targeting techniques (named here TNFR2ff mice). Mice in which exon 3 of the *Ikbkb* gene sequence is flanked by *LoxP* sites (IKKβff mice, a gift of M. Karin, Department of Pharmacology, University of California at San Diego, California, USA), have been described previously [29]. Mice with a selective depletion of TNFR1, TNFR2, or IKKβ in excitatory CNS neurons (nTNFR1KO, nTNFR2KO, nIKKβKO) were generated by crossing TNFR1ff, TNFR2ff, or IKKβff mice with mice that express a neuronal calmodulin-kinase IIa promoter-driven Cre recombinase (CamkII-Cre mice [30]). TNFR1ff, TNFR2ff, and IKKβff littermate mice were used as respective controls in all in vivo experiments.

DNA PCR analysis for the detection of Cre recombination (deletion) events in different tissues of nTNFR1KO mice was performed using 2 independent PCR reactions. Primers sense 5′-CAA GTG CTT GGG GTT CAG GG-3′ and antisense 5′-CGT CCT GGA GAA AGG GAA AG-3′ (ThermoFisher Scientific) were used for the detection of WT *Tnfrsf1a* (134-bp band) and floxed TNFR1ff alleles (195-bp band). Primers sense 5′-CCT-GCA-GAC-ACA-CGG-GGA-AA-3′ and antisense 5′-TGA-ACT-CAG-GTT-GCC-AGA-CG-3′ (ThermoFisher Scientific) were used for the detection of recombined (deleted, “defloxed”) allele (300-bp band). DNA PCR analysis for the detection of Cre recombination events in tissues of nTNFR2KO mice was performed using a combination of 3 primers in one single PCR reaction; sense 5′-CAC ATG TAT GTA CAC CTG TGT G-3′, antisense 5′-CTC TCC TGG GCC TAA TGT AG-3′, and antisense 5′-ATT GTC TAC TCA GCA CTG GG-3′ (ThermoFisher Scientific) for the detection of WT *Tnfrsf1b* allele (151-bp band), floxed TNFR2ff (200-bp band), and recombined (deleted, “defloxed”) alleles (321-bp band). Detection of Cre recombinase was performed using primers, sense 5′-ATT ACC GGT CGA TGC AAC GAG T-3′ and antisense 5′-CAG GTA TCT CTG ACC AGA GTC A-3′ (800-bp band). All mice were backcrossed and maintained on the B6 genetic background. Animals were maintained under specific pathogen-free conditions at the animal facilities of the Hellenic Pasteur Institute and all experimental procedures were approved by national authorities and conformed to ARRIVE guidelines and EU Directive 2010/63/EU for animal experiments. All experimental procedures were reviewed and approved by the Committee for Evaluation of Experimental Procedures, Department of Experimental Animal Models, Hellenic Pasteur Institute (Presided by Dr. P. Andriopoulos, pandriopoulos@patt.gov.gr, for the Hellenic Republic, General Secretariat for Agricultural Economy, Veterinary and Licenses), license numbers 4457/10-07-2014 and 2579/31-05-2018, and conformed to ARRIVE guidelines and EU Directive 2010/63/EU for animal experiments.

### Experimental autoimmune encephalomyelitis

EAE was induced in 10–12-week-old female nTNFR1KO and nTNFR2KO mice, and their respective TNFR1ff and TNFR2ff littermate controls as previously described [31]. EAE was induced by subcutaneous (s.c.) tail base injection of 37 μg of rat myelin OLG glycoprotein 35-55 peptide (MOG) dissolved in 100 μl of saline and emulsified in 100 μl complete Freund’s adjuvant (CFA) (Sigma-Aldrich) supplemented with additional 400 μg of H37Ra *Mycobacterium tuberculosis* (Sigma-Aldrich) (MOG/CFA). Mice also received an i.p. injection of 200 ng of *Bordetella pertussis* toxin (PTx) (Sigma-Aldrich) on days 0 and 2. Mice were assessed daily for clinical signs as previously described [31]. Briefly, mice were examined using an EAE scoring scale as follows: grade 0, no clinical symptom; grade 1, limp tail; grade 2, hindlimb weakness; grade 3, hindlimb paralysis; grade 4, forelimb and hindlimb paralysis; grade 5, moribund or dead. Animals with a score of 4 or above were euthanized. Mice were allowed free access to food and water throughout the experiment.

### Cuprizone-induced demyelination and remyelination

Cuprizone (CPZ) demyelination was induced in 8–10-week-old male nTNFR1KO, nTNFR2KO, and nIKKβKO mice and their respective TNFR1ff, TNFR2ff, and IKKβff littermate controls. Mice were fed ad libitum with 0.2% w/w CPZ (Sigma-Aldrich; C9012) in powdered standard mouse chow using feeder devices (Analab, Tecniplast, Italy) for 6 weeks and then returned to normal diet, as described [32]. Consistent with this model, hallmark clinical features of disease were measured in the the midline corpus callosum of CPZ-treated mice at the end of the second week of CPZ feeding (CPZ2) (microglial response), CPZ3 (microgliosis, axonal damage, and demyelination by Klüver-Barrera Luxol fast blue/ LFB staining), CPZ5 (maximal demyelination by immunohistochemical staining), and several time points after removal of CPZ from the diet at week 6 of feeding, including 1 week after cessation of CPZ feeding (CPZ6+1) (remyelination), CPZ6+2 and CPZ6+4 (both almost complete remyelination) [11].

### Acute and preconditioning kainic acid excitotoxicity models in mice

In an acute kainic acid (KA) seizure model, 2–6-month-old male nTNFR1KO and nTNFR2KO mice, and their respective TNFR1ff or TNFR2ff littermate controls, were injected intraperitoneally (i.p.) with 20 or 24 mg/kg KA according to the manufacturer’s instructions (Tocris Bioscience). In a modified preconditioning KA protocol based on a previously described model [33], mice were injected i.p. with 15 mg/kg KA and subsequently after 24 h i.p. with 20 mg/kg KA. Seizures were scored every 5 min for 90 min, using a clinical seizure scale based on previously described methods [34, 35]. Briefly, mice were scored as follows: 0, no behavioral response; 1, immobility; 2, one or more of the following: head bobbing, whiskers movement, hunching, outstretched forelimbs; 3, rearing and one-sided forelimb spasms; 4, repeated rearing and two-sided forelimb spasms; 5, one or more of the following: convulsions with sideways leaning, jumping and wild running followed by convulsions, death. Animals with a score of 5 were euthanized.

### RNA isolation and quantitative RT-PCR

The brain was removed and the spinal cord was flushed from the vertebral column after transcardial perfusion with ice-cold phosphate-buffered saline pH 7.4 (PBS) at sacrifice by carbon dioxide inhalation. Total RNA was extracted using TRIzol reagent (Thermo Fisher Scientific), according to the manufacturer’s instructions. RNA purity and quantification were assessed using NanoDrop 2000 spectrophotometer data. RNA was used for one-step quantitative RT-PCR using a QuantiFastTM SYBR® green RT-PCR kit (Qiagen Inc.) and QuantiTect Primer Assays for *Ccl2* (Mm_Ccl2_1_SG), *Cxcl16* (Mm_Cxcl16_1_SG), *H2Ab1* (Mm_H2-Ab1_1_SG), *Mbp* (Mm_Mbp_1_SG), *Olig2* (Mm_Olig2_1_SG), *Snap25* (Mm_Snap25_2_SG), *Tnf* (Mm_Tnf_1_SG), and *Gusb* (Mm_Gusb_1_SG) (Qiagen Inc). All reactions were performed in duplicate using the LightCycler system (Roche, Mannheim, Germany). At the end of each PCR run, melting curve analysis was performed to verify the integrity and homogeneity of PCR products. Results were analyzed using LightCycler software version 3.5 (Roche Diagnostics). Normalization was done using *Gusb* as a reference gene. Two methods were used for measuring RNA levels, with the same results. For spinal cord samples from EAE experiments, gene expression levels were calculated using already created standard curves for each gene as previously described [31]. These standard curves were created by plotting threshold cycle values vs the logarithm of serial diluted RNA concentrations. Least-squares methods were used for the determination of A and B values in the equation threshold cycle = A*log(CRNA) + B. The coefficient of determination (R2) was greater than 0.99. A second method was used for CPZ brain samples, except for *Snap25*, which was calculated as above. The difference between threshold cycle values of the target and reference gene (ΔCT = Ct target gene − Ct reference gene) in each sample was calculated. The relative gene expression was then estimated according to the ΔΔ threshold cycle method in which ΔΔCT = (ΔCT of a target sample) − (ΔCT of the average of reference/ naïve samples). The final relative gene expression was expressed as 2^–ΔΔCT^ value.

### Histology and immunohistochemistry

Mice were transcardially perfused with ice-cold 4% paraformaldehyde in PBS at sacrifice by carbon dioxide inhalation. For analysis of CPZ-induced pathology, brains were removed, post-fixed in the same fixative overnight at 4 °C, and embedded in paraffin. For histology and immunohistochemistry (IHC), serial coronal paraffin sections (5 μΜ) were cut through the corpus callosum corresponding to Sidman sections 295–305, and comparative analyses were made in the midline corpus callosum. Sections were stained with LFB for demyelination [11]. IHC was performed as described [11] using the primary antibodies mouse anti-2′, 3′-cyclic nucleotide 3′-phosphodiesterase (CNPase) mAb (1/700; clone SMI-91; Covance), mouse anti-amyloid precursor protein (APP) (1/350, Covance), rabbit anti–ionized calcium-binding adaptor molecule 1 (Iba1) (1/400; Wako chemicals; 019-19741), mouse anti-neurofilament H phosphorylated (SMI 31) (1/1000; clone SMI-31; Covance), rabbit anti-glial fibrillary acidic protein (GFAP) (1/300; Dako; Z0334), anti-apoptosis inducing factor (AIF), anti-CD3 (145-2C11; BD Pharmingen); followed by biotinylated secondary Abs, horseradish peroxidase-labeled avidin-biotin complex, and visualized using 3′3′-diaminobenzidine (all from Vector Laboratories). Detailed analysis of pathology in our MOG-induced EAE model has previously been described [10, 30]. Immunofluorescence staining for autophagy detection was performed as previously described [36]. Briefly, paraffin sections were immunolabeled using a polyclonal rabbit anti-mouse LC3B antibody (1:100; Sigma-Aldrich, L7543), followed by goat anti-rabbit Alexa Fluor 647 (1:400; ThermoFisher Scientific).

### Histology quantification

For histological analysis, tissue sections were viewed with an Olympus BX-50 microscope using 10x, 20x and 40x objectives, and images captured with an Olympus DP71 microscope digital camera using cell^A imaging software (Soft Imaging System GmbH). Quantitative histopathological analysis was performed in the midline corpus callosum, unless otherwise stated using the ImageJ software. Demyelination was measured as a loss of LFB staining in coronal sections of the whole corpus callosum using a semiquantitative method, as described [11, 12], and loss of CNPase immunostaining in the midline corpus callosum by densitometry. Axonal damage was measured as the numbers of positive APP-stained spheroids per mm^2^ tissue, and loss of SMI 31 immunostaining by densitometry. Microglia and astrocyte responses were measured as the area covered by Iba1- and GFAP-immunoreactivity, respectively. AIF levels were measured as the area covered by AIF-immunoreactivity, and CD3^+^ T lymphocytes were counted as cells/mm^2^. Immunofluorescence was observed and scanned using a Leica TCS-SP8 MP confocal microscope. All images were acquired as stacks of 30–33 slices imaged at 1.0-μm depth intervals using a 60x oil immersion objective. Autophagy induction was measured as the numbers of LC3B-positive puncta/LC3B-immunoreactive cell in (lateral) corpus callosum area as previously described [37], using a macro developed in Fiji software. Fifty LC3B-immunoreactive cells were analyzed for each condition (CPZ0 and CPZ5 control and nTNFR1KO mice). Excitotoxic neuronal death in the hippocampus of KA challenged mice was measured as % of pyknotic (dense Nissl-stained) cells in a set area (3.3 mm × 1.7 mm) of the affected CA3 region.

### Cortical astrocyte-neuron co-cultures

Astrocyte cultures were prepared from cortical tissues of male and female P0, based on a previously described method [38] (Supplementary Figure 4). Briefly, cells were grown in 25-cm^2^ flasks, pre-coated with poly-d-lysine (0.1 mg/ml) and laminin (20 mg/ml) (Sigma-Aldrich; P6407), in high glucose Dulbecco’s modified Eagle medium (DMEM) supplemented with 10% horse serum, 0.5 mM glutamine, and 50 U/ml penicillin-streptomycin. Cultures were grown to confluency by around 7–10 days in vitro (DIV7-10), harvested, and re-seeded on poly-d-lysine/laminin-coated glass coverslips in DMEM in 48-well plates (40.000 cells/well). They were used for co-culture with neurons on DIV5.

Cortical neurons were isolated from male and female E14.5 embryos, as previously described [39]. To prepare astrocyte-neuron co-cultures (NA), neurons were seeded onto the coverslips coated with confluent DIV5 astrocytes in 48-well plates (180.000 neurons/well) in Neurobasal medium supplemented with 2% β-27, 0.5 mΜ glutamine, and 50 U/ml penicillin-streptomycin. After 2 days of co-culture (NA-DIV2), proliferation of non-neuronal cells was halted by addition of 10 μM Ara-C for 2 days. At NA-DIV7, recombinant mouse TNF (100 ng/ml; R&D systems; 410-MT-010), or human TNF (hTNF) (100 ng/ml; R&D systems; 210-TA-010), or TNFR2 agonist (TNFR2ag) (100 ng/ml; EHD2-sc-mTNFR2 [40];), was added for 24 h. Excitotoxic death was induced in NA-DIV8 co-cultures by exposure to 50 μM N-methyl-d-aspartate (NMDA) (Sigma-Aldrich; M3262) supplemented with 10 μM glycine for 22 h, followed by fixation with 4% paraformaldehyde in PBS on NA-DIV9. The cells were immunolabeled with mouse anti-neuronal nuclear antigen (NeuN) (1/300; Chemicon; clone A60), rabbit anti- GFAP (1/400; DAKO; Z0334) followed by goat anti-mouse Alexa Fluor 488 (1/1000; Molecular Probes) or anti-rabbit Alexa Fluor 568 (1/500; Molecular Probes) antibodies and counterstained with Hoechst 33342. Immunostaining with rabbit anti-Iba1 (1/400; Wako chemicals; 019-19741), showed approximately 5% of cells in co-cultures were microglia. Excitotoxic death was measured by the percentage of Hoechst 33342-stained pyknotic nuclei in NeuN-positive neurons. Pyknotic nuclei are characteristic of neuronal necrosis induced by acute excitotoxicity in vivo and in vitro [41]. The NMDA receptor antagonist MK801 (10 μM) was added to the culture medium of control cells together with NMDA for 22 h.

Cortical neurons were also isolated from nTNFR1KO, nTNFR2KO, TNFR1ff, and TNFR2ff E14.5 embryos and used for analysis of Cre recombination. Consistent with a previous report showing that the expression of CamkII promotor-driven transgenes in mice begins after birth [42], Cre-mediated deletion of TNFR1 or TNFR2 was undetectable in embryonic neurons up to the last day of culture (DIV18).

### Statistics

Statistical analyses were performed using Graphpad Prism 7. Data are presented as mean ± SEM. Student’s t*-*test, one-way ANOVA, or Mann-Whitney was performed for pairwise comparisons between neuron viabilities as measured by Hoechst staining, relative mRNA levels, and EAE or KA scores between animals at each point in the different groups. Two-way ANOVA (Bonferroni’s test) and Student’s t*-*test were used for longitudinal analysis of CPZ experiments or pairwise comparisons at specific time points, respectively. Values of *p* ≤ 0.05 were considered statistically significant.

## Results

### Neuronal TNFR1 promotes neuroinflammation and the onset of EAE

With the aim of interpreting the opposing neurotoxic and neuroprotective effects of the TNF cytokine system described in the literature, we studied the effects of neuronal TNFR1 and TNFR2 using two CNS demyelination models in mice, EAE and CPZ-induced demyelination. We crossed TNFR1ff and TNFR2ff mice with CamkII-Cre mice to achieve TNFR1 or TNFR2 depletion selectively in glutamatergic excitatory CNS neurons. Neuron-specific TNFR1KO (nTNFR1KO) and TNFR2KO (nTNFR2KO) mice were born at the expected Mendelian ratio, were viable, were fertile, and did not display any spontaneous phenotypes up to the maximum age followed (18 months). Efficiency and specificity of TNFR1 and TNFR2 deletion in brain and spinal cord tissues was verified by DNA analysis which showed recombination specifically in brain and spinal tissues, and not any other tissue tested, in mice from 1 month of age and upwards (Supplementary Figure 1). RNA levels of TNFR1 and TNFR2 in the whole brain of adult naïve B6 control mice were detected at very low levels, and the expression of TNFR2 in nTNFR1KO brain and of TNFR1 in nTNFR2KO brain was equal to levels in respective control and B6 mice (data not shown).

nTNFR1KO, nTNFR2KO, and their respective TNFR1ff and TNFR2ff littermate controls were immunized with MOG to induce EAE, a disease driven by myelin-reactive T cells and other infiltrating immune effector cells. In our MOG-EAE model, in B6 mice, pathology is characterized by severe infiltration of the meninges and spinal cord parenchyma by immune cells (including CD4^+^ and CD8^+^ T cells, B cells, NK1.1^+^ cells, and myeloid cells), microglia and astrocyte activation, demyelination, and axonal damage in the white matter at the peak of disease. Pathology partially resolves during the chronic phase of disease [30].

**Fig. 1:**
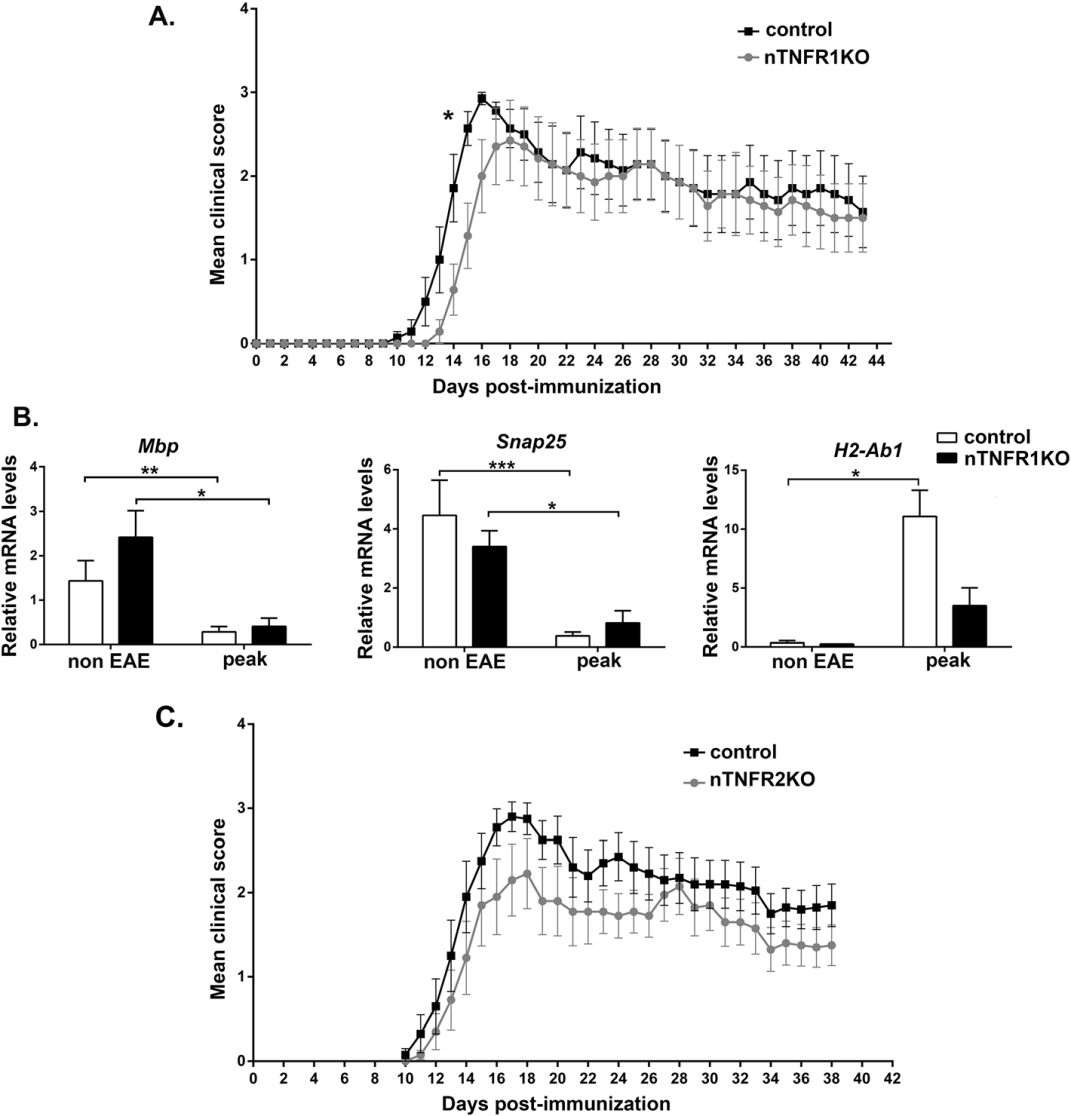
Neuronal TNFR1 advances the onset of clinical symptoms and spinal cord inflammation in EAE. (**A**) Mean clinical scores for nTNFR1KO (*n* = 7) and TNFR1ff control (*n* = 7) mice after immunization with MOG/CFA/PTx. (**B**) Differential expression of myelin, neuronal, and immune genes relative to *GusB* in total mRNA isolates taken from nTNFR1KO and control spinal cords of non-immunized (non-EAE) and immunized mice at peak of EAE in the control group (peak) (non-EAE: control, *n* = 3 and nTNFR1KO, *n* = 4; peak EAE: control, *n* = 10 and nTNFR1KO, *n* = 3). (**C**) Mean clinical scores for nTNFR2KO (*n* = 10) and TNFR2ff control (*n* = 10) mice after immunization with MOG/CFA/PTx. Results shown are representative of two (**A**) or three (**C**) independent experiments and are presented as mean values ± SEM. Statistical significance after pairwise comparisons between measurements of EAE clinical scores between the KO and control mouse strains on each day (**A**, **C**) and mRNA levels (**B**) are shown by Student’s t-test. *p ≤ 0.05, **p ≤ 0.005, ***p ≤ 0.001

nTNFR1KO, nTNFR2KO, and control mice all showed full susceptibility to EAE (Fig. 1). nTNFR1KO mice showed a small but significant delay in disease onset compared to TNFR1ff controls in 2 of 3 experiments, but thereafter the two groups of mice showed no differences in clinical disease progression up to the last time point studied (day 70) (Fig. 1A). nTNFR2KO mice developed EAE with equal onset and clinical course as TNFR2ff littermate controls up to the last time point studied (day 38) (Fig. 1C). The results suggest that neuronal TNFR1 plays a small disease-advancing role at the onset of EAE; otherwise, neuronal TNFR1 and TNFR2 do not have significant effects on the clinical course of EAE.

To investigate how neuronal TNFR1 advances EAE onset, RNA was isolated from the spinal cord of nTNFR1KO and TNFR1ff mice at disease peak in the control group and analyzed for the expression of early-disease marker genes [31]. nTNFR1KO and TNFR1ff spinal cord showed equally marked reduction in expression of myelin (*Mbp*) and neuronal (*Snap25*) gene markers compared to corresponding naïve mice confirming the onset of EAE (Fig. 1B). However, while TNFR1ff spinal cord showed significantly increased expression of the inflammatory gene marker (*H2-Ab1*) at disease peak compared to naïve, nTNFR1KO showed no increase (Fig. 1B). These results suggest that neuronal TNFR1 promotes the neuroinflammatory response in spinal cord tissue and the onset of clinical symptoms in EAE, while neuronal TNFR2 has no obvious effect on disease development.

### Neuronal TNFR1 advances the onset of microglia responses and demyelination and is necessary for OLG loss and axon damage in CPZ demyelination

We next used nTNFR1KO, nTNFR2KO, and control mice in an acute toxicity demyelination model induced by 6 weeks of dietary CPZ [32]. CPZ is a copper chelator that causes mitochondrial dysfunction and oxidative stress, both of which are key features of progressive MS [43]. In B6 mice CPZ induces acute responses of microglia and astrocytes, massive death of mature OLG between weeks 2 and 5 of CPZ feeding that is thought to be triggered by immune mechanisms [44], and predictable primary demyelination in the midline corpus callosum, followed by almost complete remyelination by 2 weeks after removal of CPZ from the diet [11, 32].

**Fig. 2:**
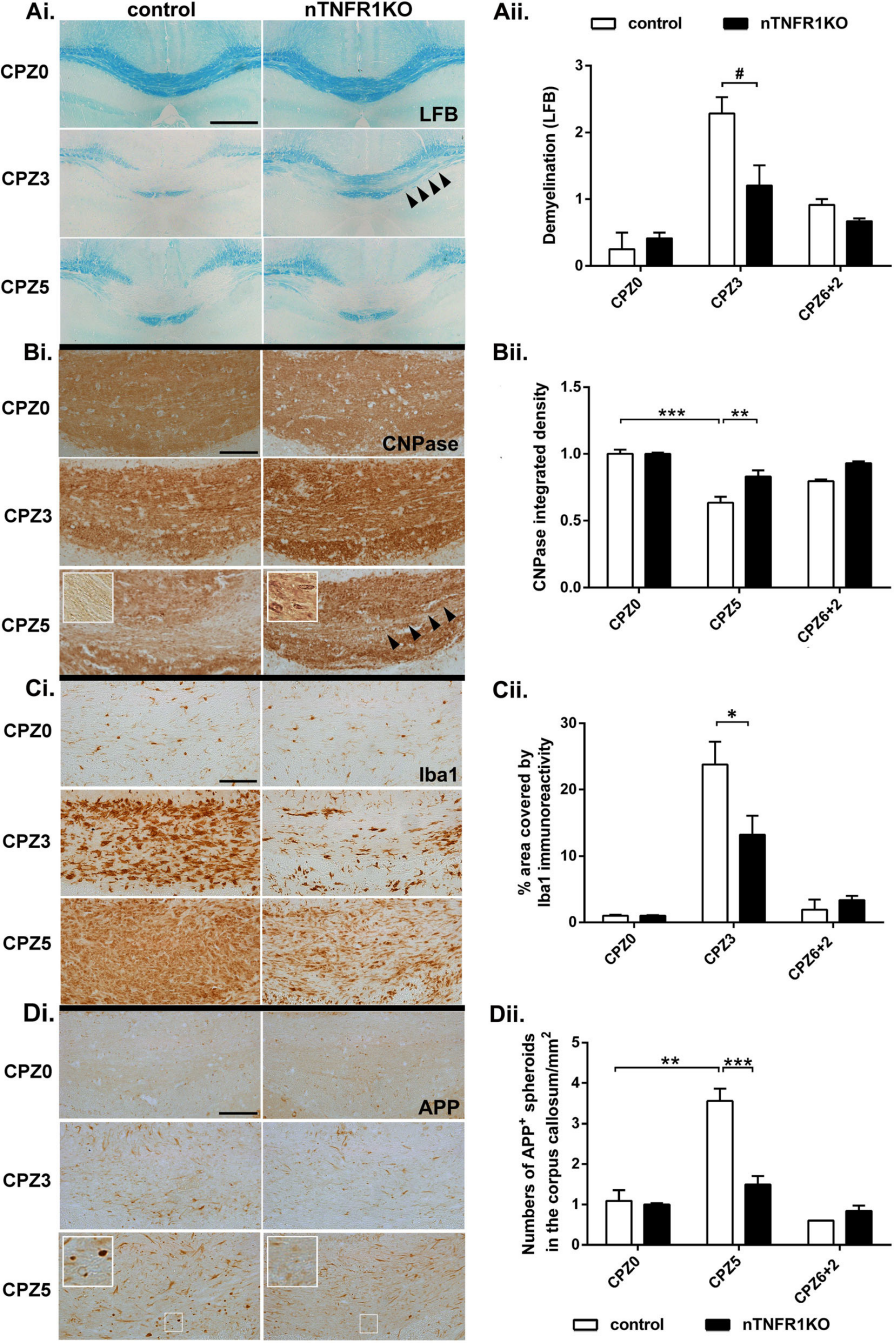
Neuronal TNFR1 promotes microglial responses, OLG loss, and axon damage in CPZ demyelination. (**Ai**) LFB staining of myelin (arrowheads showing maintenance of myelin at CPZ3 in nTNFR1KO mice), (**Bi**) CNPase immunostaining of myelin and OLG (arrowheads and inset showing maintenance of myelin and OLG at CPZ5 in nTNFR1KO mice), (**Ci**) Iba1 immunostaining of microglia, and (**Di**) APP immunostaining of axonal spheroids (inset showing higher magnification) in serial brain coronal paraffin sections through the medial corpus callosum from representative nTNFR1KO and TNFR1ff control naïve (CPZ0) or CPZ-fed CPZ3 and CPZ5 mice. Scale bars: 500 μM (Ai), and 100 μM (Bi, Ci, Di). (**Aii**) Semiquantitative scoring of demyelination (loss of LFB staining) in the medial corpus callosum of the same groups of mice represented in Ai. Quantitative representation of (**Bii**) CNPase immunoreactivity by densitometry, (**Cii**) Iba1 immunoreactivity by % area covered, and (**Dii**) numbers of APP immunoreactive spheroids/mm2 tissue in the corpus callosum of groups of mice represented in Bi, Ci, and Di, respectively. Results shown in Bii and Dii are normalized against values in CPZ0 mice of each genotype (*n* = 2 for all CPZ0 and control CPZ6+2, and n ≥ 7 mice for other time points). Results are from two independent experiments. The most relevant statistically significant differences after comparisons between groups are shown by two-way ANOVA with Bonferroni’s test (*), and pairwise comparisons by Student’s t-test (#). *, #*p* ≤ 0.05, **p ≤ 0.005, ***p ≤ 0.001

CPZ-induced pathology in the corpus callosum of nTNFR1KO mice was overall less severe than in TNFR1ff controls. nTNFR1KO mice showed less demyelination at CPZ3 by LFB staining of myelin (Fig. 2Ai and ii), a time point where myelin detected by the more sensitive CNPase immunostaining method appeared still well preserved in both strains. nTNFR1KO mice showed less OLG and myelin loss at CPZ5 by CNPase immunostaining, compared to TNFR1ff controls (Fig. 2Bi and ii). nTNFR1KO mice also showed a reduced microglia response at CPZ3 by Iba1 immunostaining (Fig. 2Ci and ii), and no measurable axon damage at any time point by numbers of axonal APP spheroids, compared to controls (Fig. 2Di and ii). Both strains showed full resolution of pathology by 2 weeks after cessation of CPZ feeding (CPZ6+2) indicating that remyelination proceeds normally. These results indicate that the combination of neuronal TNFR1 and CPZ induces stress in neurons that results in the exacerbation of CPZ-induced pathology.

**Fig. 3:**
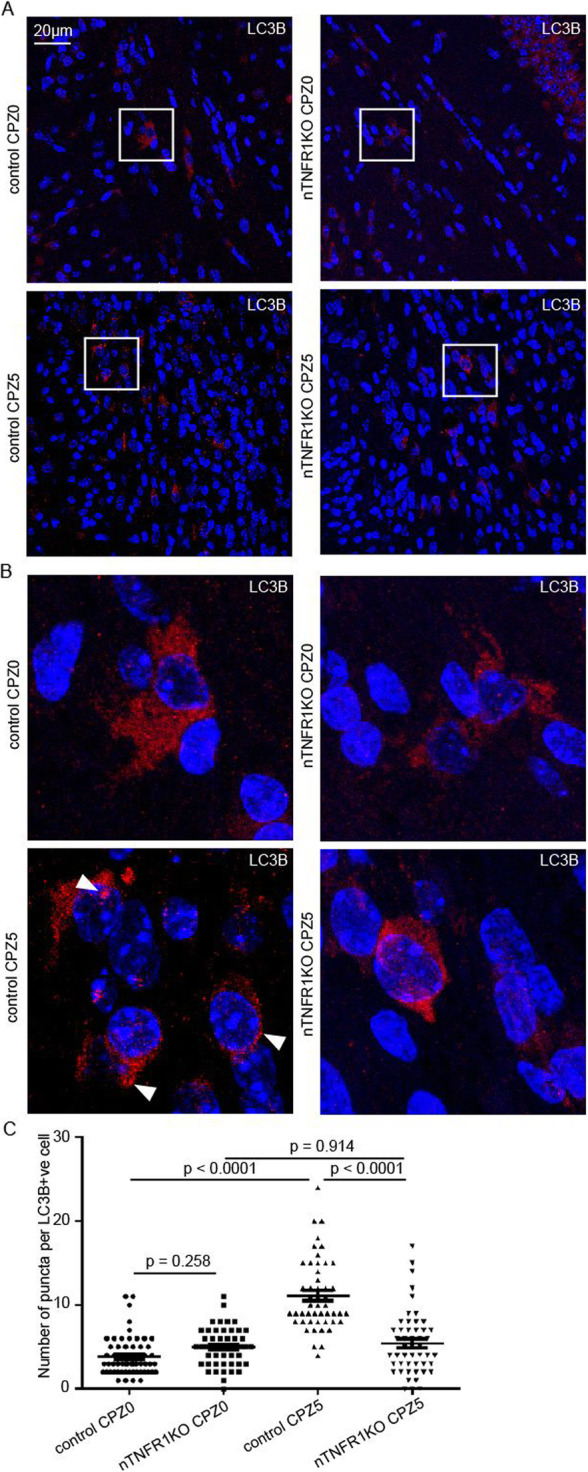
Neuronal TNFR1 increases the induction of autophagy in corpus callosum non-neuronal cells. (**A**) Immunofluorescence staining of LC3B (red), and DAPI (blue) in brain coronal paraffin sections through the corpus callosum from representative nTNFR1KO and TNFR1ff control naïve (CPZ0) and CPZ-fed CPZ5 mice (*n* = 3 for all CPZ0 and CPZ5 time points). Scale bars: 20 μm; 60x oil immersion objective. (**B**) Higher power insets from CPZ-fed CPZ5 mice of each genotype shown in (A), arrowheads showing LC3B-positive puncta. (**C**) Quantitative analysis of LC3B-positive puncta in LC3B-immunoreactive cells (*n* = 50 LC3B-immunoreactive cells were measured from each mouse strain at each time point). Means ± SD LC3B puncta/cell are depicted. p-value has been calculated by one-way ANOVA

Considering that both TNF signaling and oxidative stress are strong inducers of autophagy [45, 46], and that autophagy in microglia is involved in the degradation and clearance of myelin debris in vitro [47], we also investigated the effect of neuronal TNFR1 on autophagy in CPZ demyelination. We measured autophagy levels in the brain sections from naïve and CPZ-fed nTNFR1KO and control mice by immunostaining for LC3B, a key component of autophagosomes (Fig. 3A, insets enlarged in B, arrowheads). Autophagic induction was significantly up-regulated in cells located in the corpus callosum (non-neuronal) of CPZ5 control mice compared to naïve CPZ0 controls, measured by the numbers of LC3B-immunoreactive puncta per LC3B-positive cell (Fig. 3C). On the other hand, levels of autophagic induction were similarly low in CPZ5 and naïve CPZ0 nTNFR1KO mice, as in naïve control mice, showing an absence of upregulation (Fig. 3C). These results suggest that neuronal TNFR1 is necessary for stimulating autophagy in non-neuronal, probably glial cells populating the white matter tracts of the corpus callosum during CPZ demyelination.

nTNFR2KO and TNFR2ff mice showed no differences in CPZ-induced pathology, measured by LFB staining, or CNPase, Iba1, and APP immunostaining (Supplementary Figure 2A-D) and both strains showed full resolution of pathology by 2 weeks after cessation of CPZ feeding (CPZ6+2).

These results show that as in EAE, neuronal TNFR1 promotes neuroinflammation and demyelination in the CPZ model. Notably, neuronal TNFR1 contributes to acute microglia responses and is required for damage of both axons and OLG in this model. The effect of neuronal TNFR1 on axon damage might be direct by increasing the oxidative stress initiated by CPZ in neurons themselves, and indirect in both neurons and OLG by increasing microglia responses and the production of pro-inflammatory cytokines and chemokines. As in EAE, neuronal TNFR2 has no effect on disease development in the CPZ demyelination model.

### Neuronal IKKβ advances neuroinflammation and the onset of demyelination in CPZ demyelination

The pro-inflammatory effects of TNFR1 are dominantly mediated through activation of the transcription factor NF-κB, which in turn induces expression of genes encoding cytokines, chemokines, and anti-apoptosis molecules [22]. Under disease conditions, it is induced and is a critical mediator of inflammation. To examine the link between neuronal TNFR1 and NF-κB signaling in CNS demyelinating disease, we crossed mice carrying a conditional floxed allele for IKKβ (IKKβff mice), the main NF-κB activating kinase in the canonical NF-κB pathway [22] with CamkII-Cre mice, to generate nIKKβKO mice [30]. We previously showed that nIKKβKO mice have a small but significant delay in EAE onset similar to that described here for nTNFR1KO mice, but unlike nTNFR1KO mice, nIKKβKO mice subsequently develop a severe non-remitting disease, indicative of neuroprotective effects of neuronal IKKβ during chronic EAE [30].

**Fig. 4:**
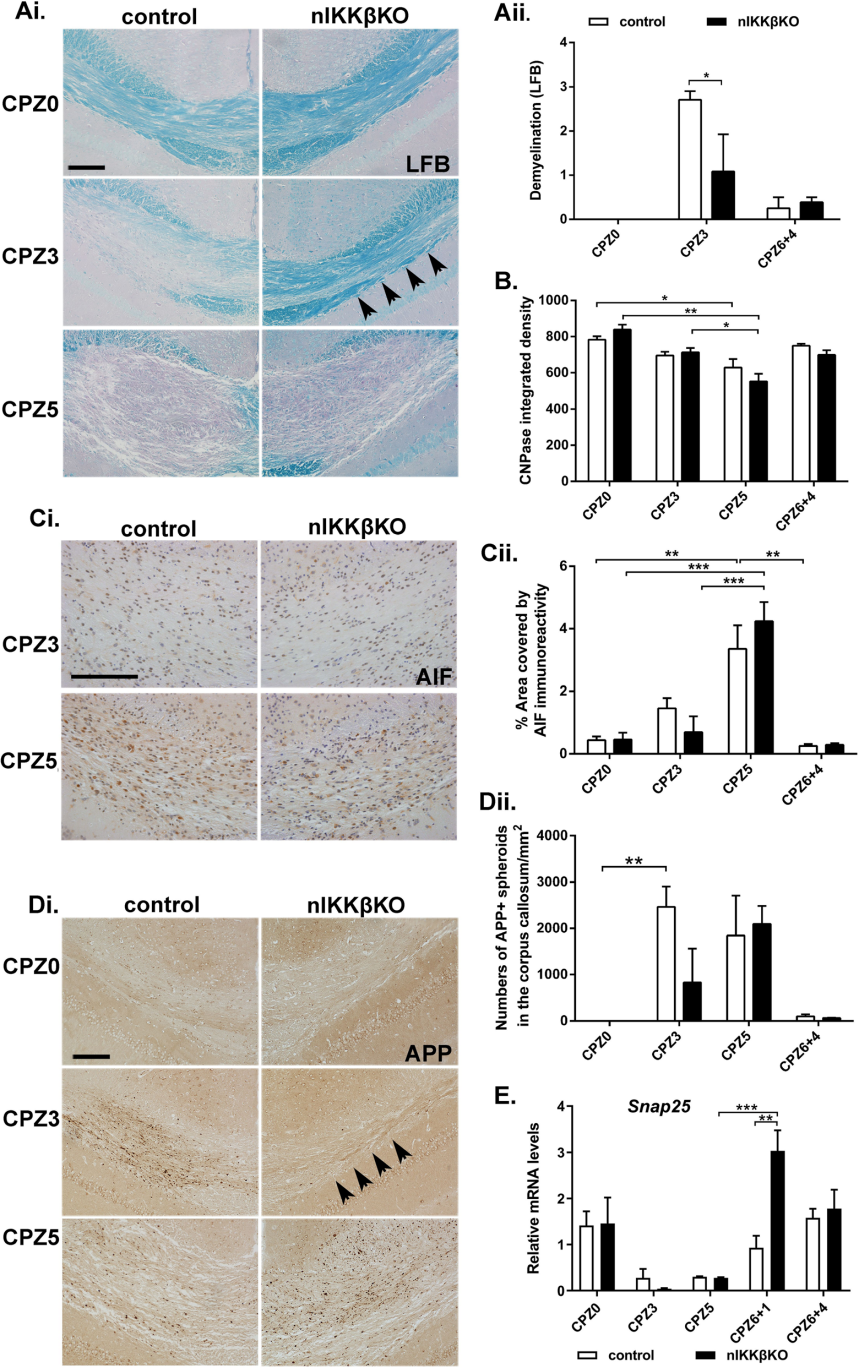
Neuronal IKKβ promotes the onset of CPZ demyelination and axon damage.(**Ai**) LFB staining of myelin (arrowheads showing maintenance of myelin at CPZ3 in nIKKβKO mice), (**Ci**) apoptosis inducing factor (AIF) immunostaining of apoptotic cells, and (**Di)** APP immunostaining of axonal spheroids in serial brain coronal paraffin sections from nIKKβKO and IKKβff control naïve (CPZ0) or CPZ-fed CPZ3 and CPZ5 mice (arrowheads showing myelin Ai reduced APP plaques Di in nIKKβKO mice at CPZ3). Scale bars: 100μΜ. (**Aii**) Semiquantitative scoring of demyelination (loss of LFB staining), (**B**) quantitative representation of CNPase immunoreactivity of myelin and OLG by densitometry, (**Cii**) AIF immunoreactivity by % area covered, and (**Dii**) numbers of APP spheroids/mm^2^ tissue in the corpus callosum in groups of nIKKβKO and IKKβff control mice represented in the photographs. (**E**) Differential expression of neuronal *Snap25* relative to *GusB* in total mRNA isolates taken from nIKKβKO and IKKβff control brains from CPZ0 or CPZ-fed CPZ3, CPZ5, CPZ6+1 and CPZ6+4 mice. Results are means of 2 (CPZ6+1) or 3-5 mice (for all other time points) from one representative of two independent experiments. The most relevant statistically significant differences after comparisons between groups are shown by two-way ANOVA with Bonferroni’s test (**Aii**, **Cii**, and **E**) and by Student’s t-test (**B** and **Dii**) *p ≤ 0.05, **p ≤ 0.005, ***p ≤ 0.001

**Fig. 5:**
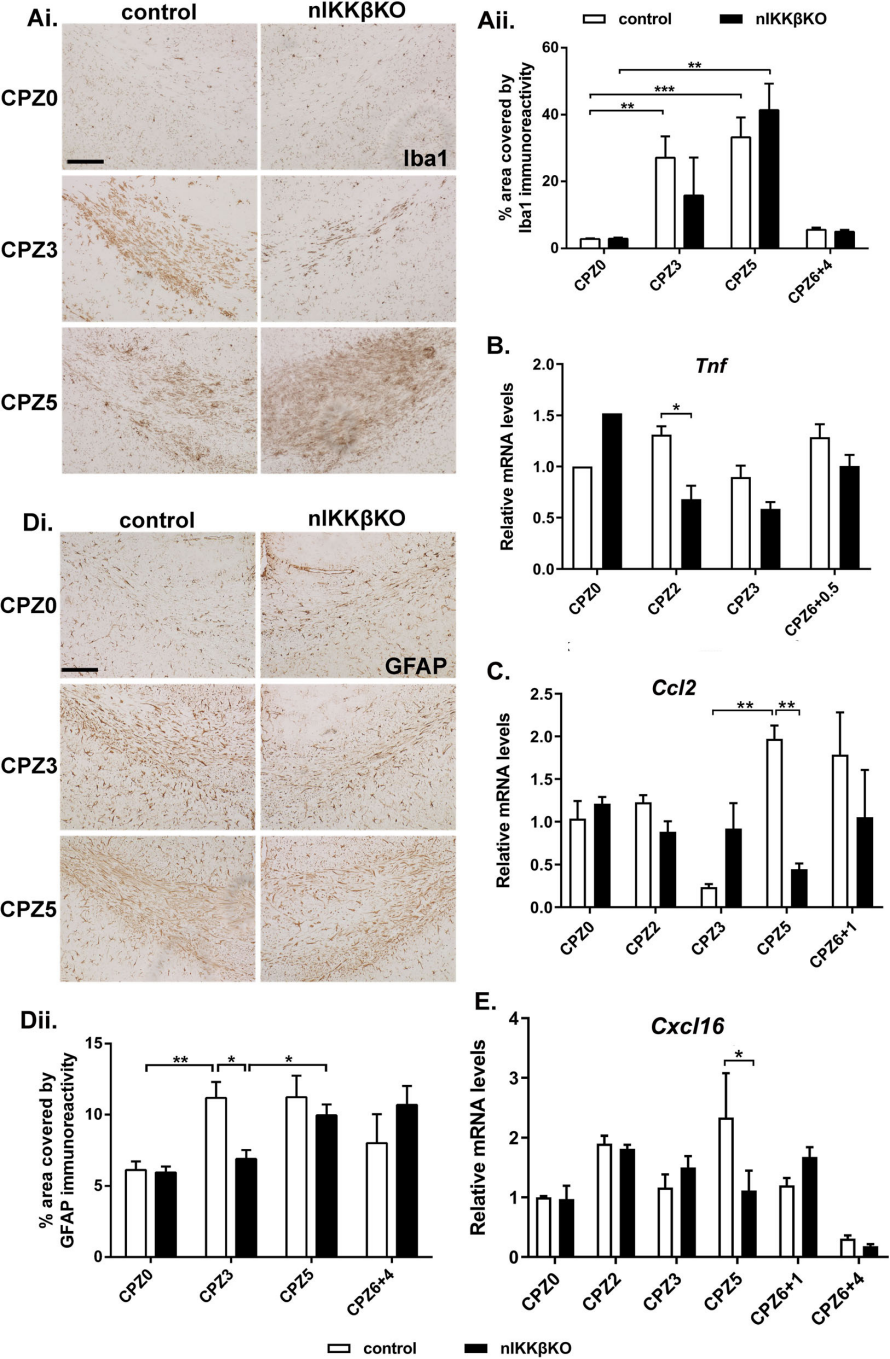
Neuronal IKKβ promotes astrocyte and microglia responses and inflammatory mediators in CPZ demyelination. (**Ai**) Iba1 immunostaining of microglia and (**Di**) GFAP immunostaining of astrocytes in serial brain coronal paraffin sections from nIKKβKO and IKKβff control naïve (CPZ0) or CPZ-fed CPZ3 and CPZ5 mice. Scale bars: 100 μM. Quantitative representation of areas covered by (**Aii**) Iba1 immunoreactivity and (**Dii**) GFAP immunoreactivity in the medial corpus callosum in groups of nIKKβKO and IKKβff control mice represented in the photographs. (**B**,** C**, and **E**) Differential expression of the inflammatory marker genes *Tnf*, *Ccl2*, and *Cxcl16* relative to *GusB* in total mRNA isolates taken from nIKKβKO and IKKβff control brains from CPZ0 or CPZ-fed CPZ2, CPZ3, CPZ5, CPZ6+0.5, CPZ6+1, and CPZ6+4 mice. Results are means of 2 (nIKKβKO CPZ6+0.5, CPZ6+1, nIKKβKO CPZ6+4) or 3–5 mice (for all other groups and time points) from one representative of two independent experiments. The most relevant statistically significant differences after comparisons between groups are shown by Student’s t-test (Aii and Dii) and by two-way ANOVA with Bonferroni’s test (B, C, and E). *p ≤ 0.05, **p ≤ 0.005, ***p ≤ 0.001

Here, we induced CPZ demyelination in nIKKβKO and IKKβff mice. At the onset of pathology at CPZ3, nIKKβKO mice showed reduced demyelination by LFB staining of myelin (Fig. 4Ai and ii), and GFAP immunostaining of astrocytes (Fig. 5Di and ii) compared to IKKβff controls, effects that resemble those in nTNFR1KO mice. In addition, axon damage by numbers of APP-immunoreactive spheroids in axons (Fig. 4Di and ii) and neuroinflammation by Iba1 immunostaining of microglia (Fig. 5Ai and ii) was increased at CPZ3 compared to naïve CPZ0 animals in IKKβff control mice, but not in nIKKβKO mice. Unlike nTNFR1KO mice, nIKKβKO mice subsequently developed full pathology with loss of myelin and OLG measured at CPZ5 by CNPase immunostaining (Fig. 4B). This was further shown by immunostaining for apoptosis-inducing factor (AIF), a mitochondrial oxidoreductase that contributes to apoptosis and has been implicated in CPZ-induced OLG death [44]. AIF-immunoreactivity was increased in the corpus callosum of both IKKβff and nIKKβKO mice at CPZ5, most probably in OLG which are cells that undergo apoptosis in this model (Fig. 4Ci and ii).

Analysis of whole brain RNA levels for disease marker genes, first for myelin, showed that naïve (CPZ0) nIKKβKO mice express higher levels of *Mbp* in the brain compared to IKKβff mice (Supplementary Figure 3A). Expression of *Mbp* and *Olig2* dropped during CPZ feeding and increased again in both nIKKβKO and IKKβff mice after CPZ removal, showing myelin recovery independently of neuronal IKKβ (Supplementary Figure 3A, B). Expression of the neuron-specific gene *Snap25*, which encodes a protein essential for synaptic function, sharply dropped during CPZ feeding and increased again in both groups after CPZ removal. Interestingly, the rebound increase of *Snap25* expression in nIKKβKO mice was consistently much higher than in controls (Fig. 4E), suggesting that neuronal NF-κB might play a regulatory role in the recovery of neuronal functions following CPZ toxicity. Expression of inflammatory markers *Tnf*, *Ccl2*, and *Cxcl16* in control IKKβff mice showed increases at two distinct time points, first at CPZ2 at the initiation of microglia responses (*Tnf*, *Ccl2*, *Cxcl16*) and again at CPZ5 at the peak of demyelination/initiation of remyelination (*Ccl2*, *Cxcl16*), or CPZ6+0,5 during remyelination (*Tnf*) (Fig. 5B, C, and E). Neither *Tnf* nor *Ccl2* showed an initial increase in expression at CPZ2 in nIKKβKO mice, correlating with the delay in glial cell activation observed in these mice at CPZ3 (Fig. 5B, C). Also, levels of *Ccl2* and *Cxcl16* were significantly lower in nIKKβKO mice at the peak of disease at CPZ5 (Fig. 5C, E). These results support the pathological findings that neuronal IKKβ promotes neuroinflammation induced by CPZ feeding.

T cells, and specifically IL-17-producing T cells, are reported to participate in CPZ demyelination [48], so we investigated T cell infiltration by counting numbers of CD3-immunoreactive cells in the corpus callosum. Infiltrating CD3-immunoreactive T cells were localized in the corpus callosum of control IKKβff mice at CPZ5, and infiltration was resolved after CPZ withdrawal at CPZ6+1 and CPZ6+4 (Supplementary Figure 3 Ci&ii). Infiltrating CD3-immunoreactive T cells were also localized in the corpus callosum of nIKKβKO mice but, unlike in control IKKβff mice, continued to accumulate after CPZ withdrawal at CPZ6+1, showing late resolution at CPZ6+4 (Supplementary Figure 3 Ci&ii).

These data show that neuronal NF-κB activity, like neuronal TNFR1, plays a role in initiating neuroinflammation (microglia and astrocytes), demyelination and axon damage in response to dietary CPZ, and might have additional, different roles to TNFR1 during disease resolution and recovery.

### Neuronal TNFR2 increases preconditioning protection against seizures and the survival of hippocampal neurons in KA excitotoxicity

Evidence for neuroprotective properties of TNF stems mainly from in vitro studies in which solTNF pretreatment of enriched neuron cultures (TNF preconditioning) protects them against a wide range of metabolic, excitotoxic, and oxidative death stimuli and promotes maintenance of calcium homeostasis [14, 15]. To investigate the role of neuronal TNFR1 or TNFR2 in glutamate excitotoxicity in vivo, we first used an acute KA excitotoxicity model. KA is a non-degradable glutamate analogue that induces epileptic seizures and death of CA3/CA2 hippocampal neurons in susceptible mouse strains. Notably, B6 mice are known to exhibit high seizure scores but no excitotoxic neuron death [34]. Also, low doses of KA are known to protect mice against seizures induced by subsequent higher doses of KA [33], allowing us also to examine the effect of neuronal TNFR in preconditioning protection against glutamate excitotoxicity in vivo.

**Fig. 6:**
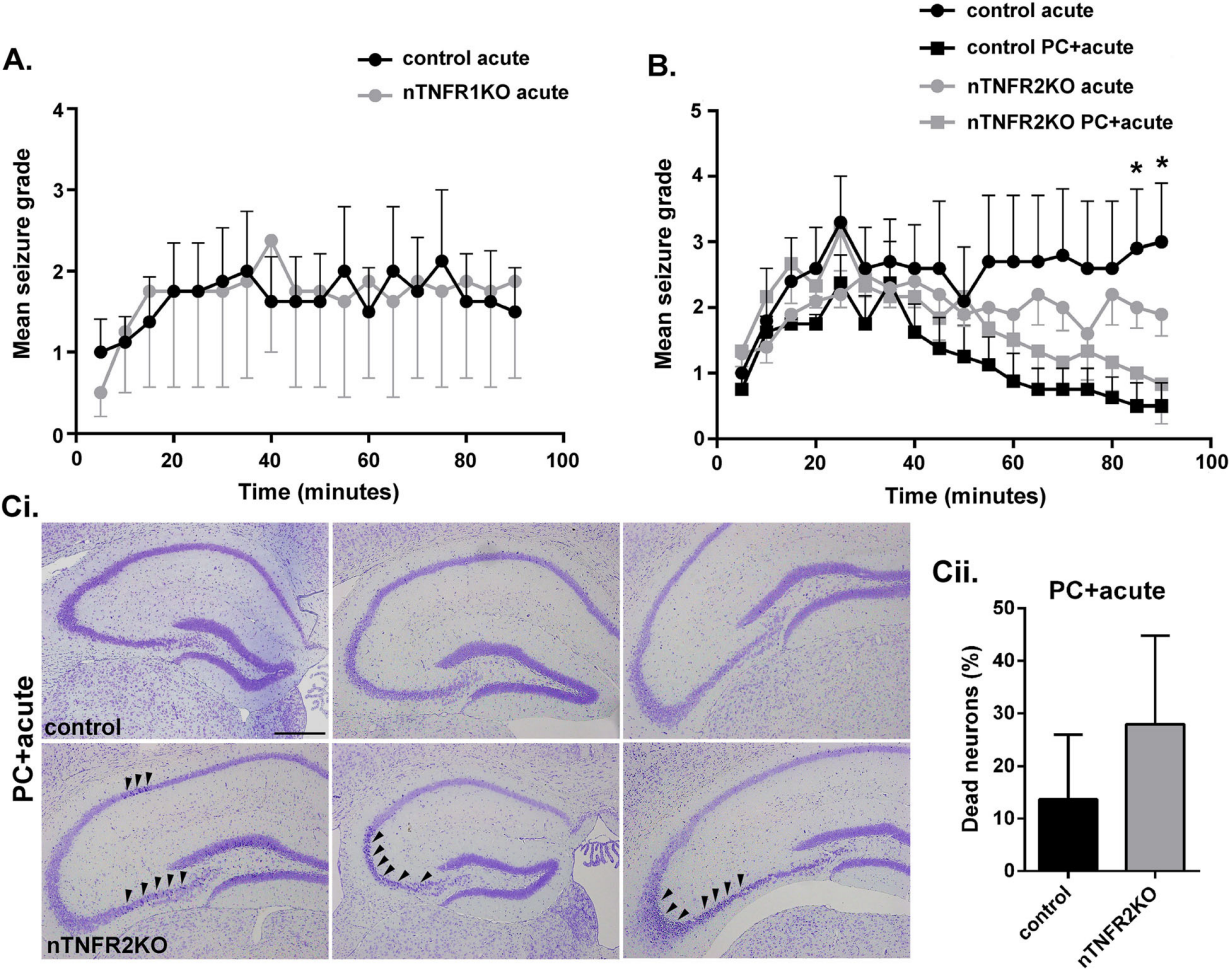
Preconditioning protection against KA seizures and hippocampal neuron survival is reduced in nTNFR2KO mice. (**A**) Mean seizure scores recorded every 5 min after acute i.p. injection of 24 mg/kg KA in nTNFR1KO (*n* = 4; gray circles) and TNFR1ff control (*n* = 4; black circles) mice. (**B**) Mean seizure scores recorded every 5 min after acute i.p. injection of 20 mg/kg KA in nTNFR2KO (*n* = 5; gray circles) and TNFR2ff control (*n* = 5; black circles) mice, or after preconditioning with i.p injection of 15 mg/kg KA followed after 24 h by i.p. injection of 20 mg/kg KA in TNFR2KO (*n* = 4; gray squares) and TNFR2ff control (*n* = 4; black squares) mice. (**C**) Cresyl violet (Nissl) staining of brain coronal paraffin sections from mice shown in (B) taken 5 days after acute KA administration in preconditioned (PC+acute) mice. (**Ci**) Low power photographs showing the variable localization of pyknotic dead neurons in the CA1, CA2, and CA3 regions of the hippocampus from nTNFR2KO mice (arrowheads). (**Cii**) Quantification of pyknotic dead neurons in mice showing an affected CA3 region, as mean % dead cells/ set area of CA3 (3.3 mm × 1.7 mm). Scale bar: 200 μΜ. Results are representative of one or two (acute KA in nTNFR2KO and TNFR2ff control mice) independent experiments and are presented as mean values ± SEM. Statistical significance after comparisons between acute KA versus preconditioning KA in each genotype is shown by one-way ANOVA with Bonferroni’s test for each time point. *p ≤ 0.05

Acute systemic (i.p.) administration of KA (20 or 24 mg/kg) consistently induced seizures in groups of nTNFR1KO, nTNFR2KO, and control mice without high mortality. No differences in seizure activity between nTNFR1KO (Fig. 6A, gray circles) and TNFR1ff controls (Fig. 6A, black circles) or nTNFR2KO (Fig. 6B, gray circles) and TNFR2ff controls (Fig. 6B, black circles) were observed in this acute model during the 90 min of monitoring, although a trend for less severe scores was noted in nTNFR2KO mice compared to TNFR2ff controls (Fig. 6B).

We next used a KA preconditioning protocol, adapted from one previously described [33]. In TNFR2ff controls low dosage KA (15 mg/kg, i.p.) significantly reduced severity of seizures induced by subsequent high dosage KA (20 mg/kg, i.p.) (Fig. 6B, black squares) compared to high dosage KA alone (Fig. 6B, black circles), an effect consistent with KA preconditioning protection. In nTNFR2KO mice, however, the preconditioning protection effect of low dosage KA on seizures induced by subsequent high dosage KA (Fig. 6B, gray squares) compared to high dosage KA alone (Fig. 6B, gray circles) was reduced and non-significant. The loss of KA preconditioning protection in nTNFR2KO mice appeared to be a combined effect of reduced acute seizure intensity and reduced preconditioning protection, but these results provide the first indication that neuronal TNFR2 is necessary for effective preconditioning neuroprotection induced by low-dose KA during glutamate excitotoxicity in vivo. A direct protective role for neuronal TNFR2 was further supported by the presence of patches of pyknotic neurons stained by cresyl violet (Nissl), a feature of neuron death, within CA1, CA2, and CA3 regions of the hippocampus of nTNFR2KO, but not TNFR2ff mice, 5 days after KA seizures with preconditioning (Fig. 6C). The patches of dead neurons in the nTNFR2KO animals were variable in location and distribution (Fig. 6Ci), not only between different animals in the same group but also between the two hemispheres of the same animal. This variability made quantification of the results challenging and differences between nTNFR2KO and TNFR2ff controls, while showing a trend towards increased death in the CA3 region of nTNFR2KO mice, did not reach statistical significance (Fig. 6Cii).

### Astrocyte TNFR1 and tmTNF via neuronal TNFR2 are necessary for, and a TNFR2 agonist reproduces, TNF preconditioning protection of cortical neurons against NMDA excitotoxicity in vitro

To reconcile previously reported neuroprotective effects of solTNF (and therefore TNFR1) against glutamate excitotoxicity in vitro [14, 15], with the in vivo findings of TNFR2 neuroprotection here, we modeled different cellular TNF/TNFR interactions in vitro using astrocyte-neuron co-cultures and measured glutamate excitotoxicity induced by NMDA in neurons. Mouse cortical neurons and astrocytes were isolated from WT mice, or mice deficient in TNF (TNFKO), solTNF (tmTNFKI), TNFR1 (TNFR1KO), or TNFR2 (TNFR2KO), and different combinations of these cells were used for co-cultures.

In this system, exposure of WT astrocyte-neuron co-cultures to NMDA (50 μM) supplemented with glycine (10 μM) for 24 h induced neuron death, measured by increased percentage of NeuN-positive neurons with pyknotic nuclei stained by Hoechst (Supplementary Figure 4). Astrocyte viability was not affected by NMDA (data not shown), and neuron death was strongly inhibited by the non-competitive NMDA antagonist MK801 (Supplementary Figure 4). Consistent with previous studies [10, 14], pretreatment of WT B6 neuron-astrocyte co-cultures with soluble recombinant hTNF or mTNF (100 ng/ml) for 24 h prior to NMDA challenge, consistently and equally protected neurons by 17–20% against NMDA death, and hTNF was used for all experiments described below (Supplementary Figure 4; Fig. 6).

**Fig. 7:**
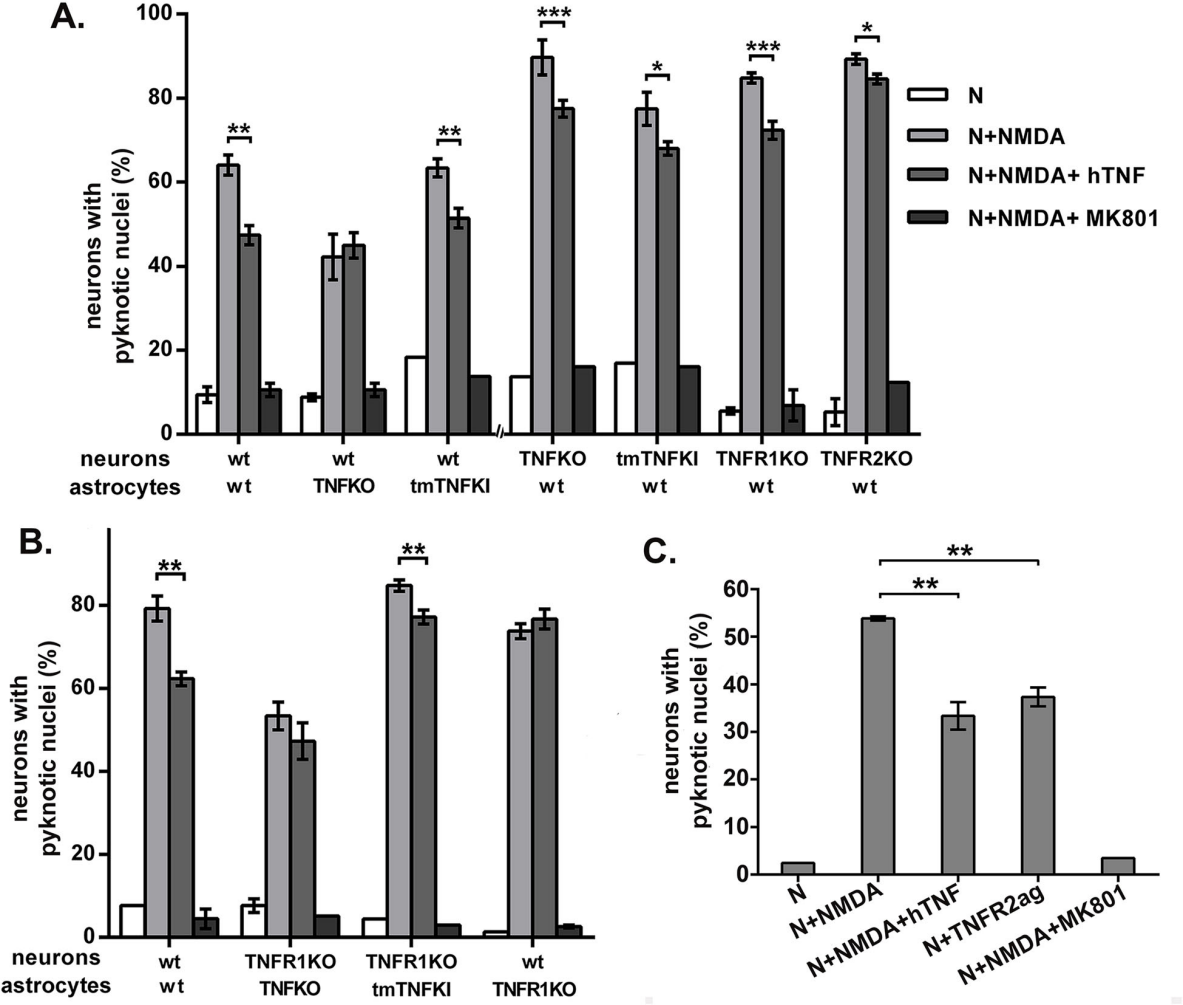
Astrocyte tmTNF-neuronal TNFR2 interactions are necessary for TNF neuroprotection against NMDA excitotoxicity in vitro. (**A** and **B**) Addition of hTNF in astrocyte-neuron co-cultures made from different combinations of WT, TNFKO, and TNFRKO mice 24 h before NMDA-induced cell death. Specifically, neuron-astrocyte co-cultures at day in vitro 7 of co-culture (NA-DIV7) were pretreated with 100 ng/ml hTNF for 24 h, NMDA excitotoxic death was induced with 50 μΜ NMDA/10 μΜ glycine on NA-DIV8, and death was measured after 22 h at NA-DIV9. (**C**) Addition of a TNFR2ag in NA-DIV7 co-cultures made from WT mice 24 h before NMDA-induced excitotoxicity provides equal neuroprotection against NMDA-induced cell death as hTNF. As control, NMDA-induced neuronal death was completely inhibited by pretreatment with the NMDA receptor antagonist MK801. Results shown are means ± SEM of duplicate or triplicate samples from one representative of two or three independent experiments. *p ≤ 0.05, **p ≤ 0.005, ***p ≤ 0.001, for pairwise comparisons between cells in each genotype combination by Student’s t-test

Neuroprotection by hTNF was absent when WT neurons were cultured with TNFKO astrocytes but maintained in WT neurons cultured with tmTNFKI astrocytes, and in TNFKO and tmTNFKI, as well as TNFR1KO neurons cultured with WT astrocytes (Fig. 7A). Importantly, neuroprotection was reduced when TNFR2KO neurons were cultured with WT astrocytes (Fig. 7A). These results suggest that preconditioning protection of cortical neurons induced by exogenous soluble hTNF is mainly dependent on astrocyte tmTNF-neuronal TNFR2 interactions. Under these conditions, neuronal TNFR1 also has a very small neuroprotective effect, detectable in WT astrocyte- TNFR2KO neuron co-cultures (Fig. 7A).

To understand how soluble hTNF triggers this mechanism, we performed experiments using TNFR1KO neurons and astrocytes. hTNF signals efficiently through mouse TNFR1 but not through TNFR2, due to species specificity of mouse TNFR2 [49]. The absence of TNFR1 from astrocytes, not neurons, abolished protection (Fig. 7B). Together, the results suggest that soluble hTNF engages astrocyte TNFR1, inducing activation and production of tmTNF. solTNF has been previously shown to increase the expression of tmTNF in cell lines [50]. Astrocyte tmTNF-neuronal TNFR2 interactions then precondition neurons for protection against glutamate excitotoxicity.

To confirm the neuroprotective role of neuronal TNFR2, we compared pretreatment of WT astrocyte-neuron co-cultures at NA-DIV7 with a novel TNFR2 agonist (TNFR2ag 100 ng/ml) [40], or with soluble hTNF (100 ng/ml), for 24 h prior to NMDA challenge. As previously described [40, 51], TNFR2ag pretreatment induced strong neuroprotection against NMDA death, equal to that induced by soluble hTNF (Fig. 7C). Together, these results suggest that astrocyte tmTNF-neuronal TNFR2 interactions dominantly mediate preconditioning protection of neurons against glutamate excitotoxicity in vitro, and likely in vivo. Neuronal TNFR1 has limited neuroprotective effect in models of glutamate excitotoxicity.

## Discussion

The TNF cytokine system is complex and associated with multiple functions in the CNS. It mediates overall neurotoxic and neuroprotective effects during CNS pathology depending on the cellular and molecular specificities of ligand-receptor interaction, and the underlying pathological mechanisms involved [1, 2]. While the main effects of TNF in the CNS are undoubtedly mediated by non-neuronal cells including resident glia and infiltrating immune cells during disease, here we wanted to investigate the specific contributions of neuronal TNFR1 and TNFR2 to inflammatory and excitotoxic neurodegeneration processes relevant for MS, and to place these in the context of previous knowledge concerning neuroprotective effects of solTNF (and therefore TNFR1) in neuronal cultures in vitro [14, 15]. We used mice in which TNFR1, TNFR2, or IKKβ was selectively depleted from excitatory glutamatergic neurons for experimental models of CNS demyelination and excitotoxicity, and astrocyte-neuron co-culture systems with different combinations of WT, TNFKO, tmTNFKI, TNFR1KO, and TNFR2KO cells in a glutamate excitotoxicity model.

The TNF system has well-described inflammatory and neurotoxic effects in the CNS. In vivo studies using conventional TNF or TNFR knockout mice, or TNF inhibitors, showed neurotoxic roles for solTNF and TNFR1 in a wide range of experimental models including ischemia-reperfusion retinal damage [17], spinal cord injury [52], Parkinson’s disease [53], Alzheimer’s disease [54], and MS [18, 19]. However, when TNFR1 is absent or solTNF is blocked in the entire organism, it is not possible to interpret the cellular mechanisms involved in neurotoxicity. A study of bone marrow chimeric mice generated using WT and TNFR1KO bone marrow cells showed the essential TNFR1-responsive cells during EAE induction are CNS-resident cells [55]. Potent inflammatory effects of TNF, directly by activation of microglia, astrocytes, and endothelial cells, and indirectly through the induction of cell necroptosis and further neuroinflammation [56], are likely to dominate the neurotoxic effects of TNF in vivo. This is supported by the finding that NF-κB activation in nestin-expressing cells (astrocytes and neurons), and not OLG, determines neuroinflammation and myelin damage in the CPZ model [57].

Here we show that both neuronal TNFR1 and IKKβ accelerate microglia and astrocyte responses, demyelination, and the onset of axon damage in the demyelination models, indicating common signaling pathways. Neuronal TNFR1 also induced the induction of autophagy in non-neuronal cells in the demyelinating lesions in the CPZ model, the identity of which remains to be determined. Interestingly, autophagy was recently found to be involved in microglial autophagy-associated phagocytosis and recovery in EAE [47] and it is possible that autophagy activation is upregulated in myelin-phagocytosing microglia present in lesions during CPZ demyelination in TNFR1ff mice. Axon damage and OLG loss were dependent upon the presence of neuronal TNFR1, not neuronal IKKβ during CPZ demyelination, suggesting that other TNFR1 pathways signal axon and OLG pathology in this model. Several known TNF effects on neurons might be involved. First, by regulation of neuronal glutamate receptors. solTNF via TNFR1 rapidly (15 min) increases cell surface levels of Ca^++^-permeable GluR1-containing α-amino-3-hydroxy-5-methyl-4-isoxazolepropionic acid (AMPA) receptor in mouse hippocampal neurons, thereby enhancing synaptic strength at excitatory synapses in vitro [58], and potentiating excitotoxicity in vivo [59–61]. Similar effects are seen with NMDA receptors [62, 63]. Glutamate receptors and transporters are dysregulated during experimental demyelination in vivo [64] and might be sufficient to activate microglia in vivo in the context of disease and induce neuroinflammation. Second, by the induction of oxidative stress in neurons. Exposure of neurons to pathophysiological concentrations of solTNF results in a rapid and profound decrease in mitochondrial function and reduced viability, an effect reduced by pretreatment of neurons with an anti-TNFR1 antibody [65]. In the CPZ toxicity model, the function of many enzymes and mitochondria is already compromised by copper chelation, resulting in metabolic stress and the selective loss of OLG and demyelination [44]. Like OLG, neurons are also highly sensitive to oxidative stress. Damage of neuronal axons is a pathological feature of acute CPZ toxicity in young B6 mice and observed here in TNFR1ff control mice by increased numbers of APP-immunoreactive spheroids at the peak of demyelination. The observation that APP spheroids do not accumulate in nTNFR1KO mice indicates that the combination of neuronal TNFR1 and CPZ causes direct damage in neurons and their axons.

The TNF system also has prominent neuroprotective roles in the CNS. One of the first reported effects of solTNF (and therefore TNFR1) in vitro was neuroprotection. Pretreatment of primary neuron cultures with solTNF protected them against a variety of metabolic-excitotoxic-oxidative insults and promoted maintenance of calcium homeostasis [14, 15]. Neuroprotective effects of TNFR1 and TNFR2 were also observed in specific in vivo experimental paradigms including cerebral stroke [16] and glutamate excitotoxicity [39, 51], but the cellular mechanisms of these effects are poorly understood. Recently, the availability of mouse lines carrying conditional TNFR1 [27] or TNFR2 [28] alleles made investigation of cell-specific roles of the TNFR in CNS possible. An elegant study using mice with myeloid cell-specific ablation of TNFR2 shows that microglial TNFR2 protects mice against the initiation of EAE [20]. Another study using mice with OLG lineage cell-specific ablation of TNFR2 shows that OLG TNFR2 ameliorates clinical symptoms particularly in the chronic phase of EAE, reduces myelin and axonal pathology, drives the differentiation of OLG precursor cells into mature OLG, and improves remyelination [21]. The effects of OLG-specific TNFR2 depletion in EAE largely recapitulate the effects of total TNFR2KO in CPZ demyelination [12] suggesting that protective effects of TNFR2 in models of inflammatory demyelination are mediated predominantly by microglia and OLG. The finding here that neuronal TNFR2 did not alter the development of EAE or CPZ demyelination further supports this conclusion. Neuronal TNFR2 did however play a role in the induction of preconditioning protection against seizures in a KA excitotoxicity model. Also, neuronal TNFR2 protected hippocampal neurons against excitotoxic death in control mice.

Together, our results show that neuronal TNFR1 and IKKβ promote neuroinflammation and the onset of demyelination in mouse models of MS and that neuronal TNFR1 is additionally required for axon damage, OLG loss, and induction of glial cell autophagy in white matter lesions during CPZ demyelination, possibly by increasing neuronal oxidative stress. On the contrary, neuronal TNFR2 had no detectable effects on the clinical or histopathological features of the MS models, but our results provide the first evidence that TNFR2 directly protects neurons in vivo under conditions of glutamate excitotoxicity that are relevant for a wide range of CNS diseases. Also, the previously reported neuroprotective effects of solTNF (and therefore TNFR1) in vitro were reconciled here with in vivo findings, with evidence from astrocyte-neuron co-cultures that astrocyte TNFR1 and tmTNF, and neuronal TNFR2 are necessary, and that a TNFR2 agonist is sufficient, to mediate TNF preconditioning neuroprotection against glutamate excitotoxicity in vitro. This is consistent with previous findings with TNFR2 agonists, which protected human dopaminergic neuronal cells against oxidative stress via activation of the PI3K-PKB/Akt pathway, or challenge with 6-OHDA in vitro [66], and enriched mouse cortical neurons against glutamate excitotoxicity (51).

## Conclusions

**Fig. 8:**
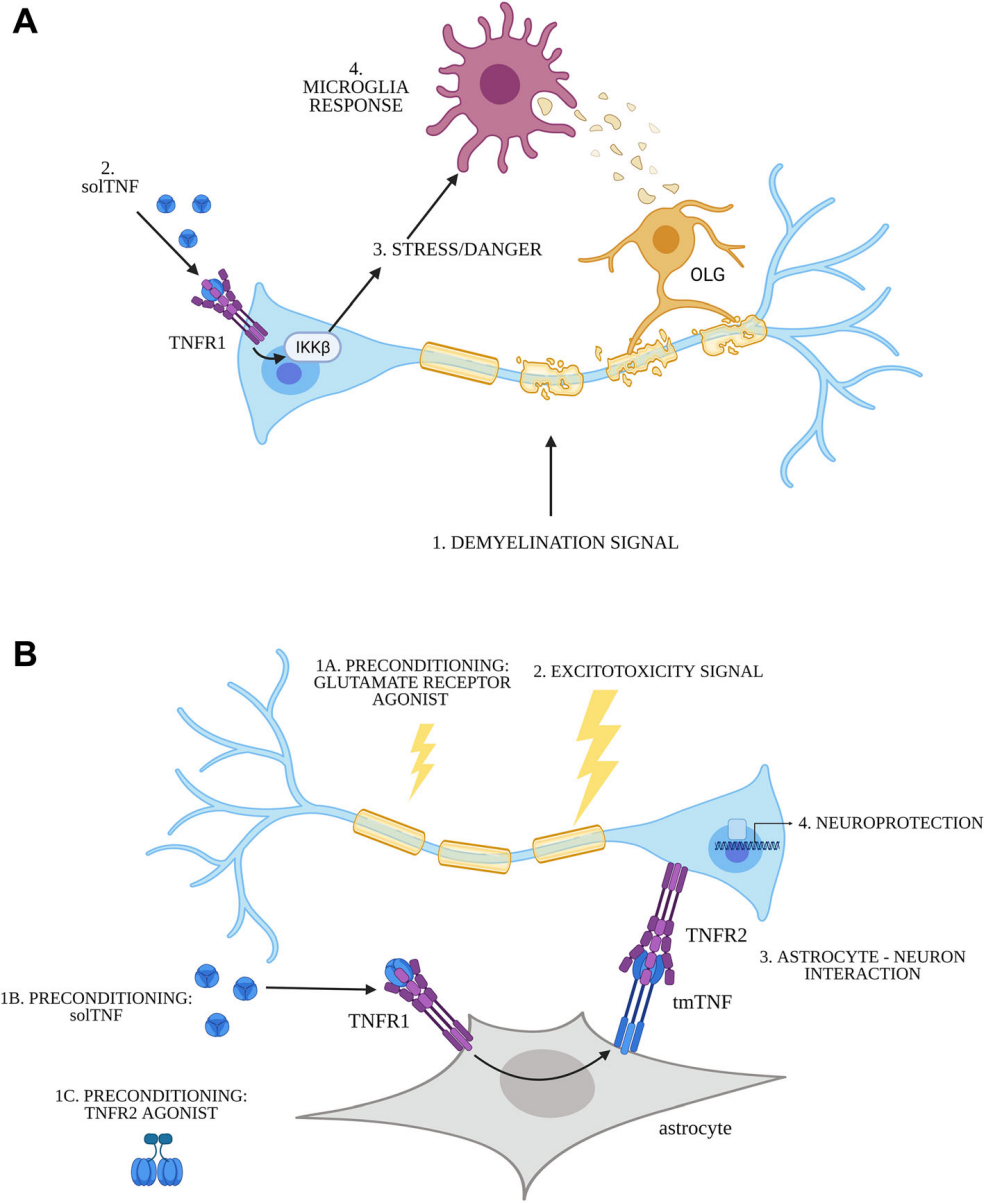
Schematic representation of the different roles of neuronal TNFR in CNS pathology described in the present study. (**A**) Neuronal TNFR1 and IKKβ promote neuroinflammation, demyelination, and axonal damage in the context of demyelination (1) signaled by EAE or CPZ toxicity. solTNF present in the microenvironment of the inflammatory lesion (2) engages TNFR1 present on the surface of neurons (as well as other cell types) and, either directly through a neuronal stress response (3), or indirectly by increasing OLG damage, enhances microglia responses and further promotes solTNF secretion and inflammation (4). (**B**) Neuronal TNFR2 is necessary for preconditioning neuroprotection against glutamate excitotoxicity. Preconditioning of neurons by challenge with low-dose KA (in vivo, 1a), preincubation with solTNF (in vitro, 1b), or preincubation with a TNFR2 agonist (in vitro, 1c) protects them against subsequent glutamate excitotoxicity (2) induced by high dose KA (in vivo) or NMDA (in vitro). In vitro experiments using TNF and TNFR mutant cells revealed that astrocytes can mediate the preconditioning effect of solTNF, via TNFR1 and expression of tmTNF, which in turn is necessary for interaction with neuronal TNFR2 (3) and the induction of neuroprotection mechanisms (4). Ligation of neuronal TNFR2 by the TNFR2 agonist (1c) is sufficient to reproduce this effect in vitro

The results of this study show that the neuronal TNFR have fundamentally different roles in CNS pathology in vivo, with signaling through neuronal TNFR1 and IKKβ promoting microglia responses and neurotoxicity in mouse demyelination models (Fig. 8A), and neuronal TNFR2 being involved in preconditioning protection in excitotoxicity, but not in inflammatory demyelination (Fig. 8B). Details of the molecular and cellular mechanisms underlying previously described neuroprotective properties of solTNF in vitro were obtained using astrocyte-neuron co-cultures with different combinations of WT and KO cells. Results further supported the in vivo findings, with solTNF (and therefore TNFR1) indirectly protecting neurons against NMDA excitotoxicity via astrocyte tmTNF- neuronal TNFR2 interactions and being fully reproduced by a TNFR2 agonist (Fig. 8B). Together the results consolidate the concept that selective inhibition of solTNF/TNFR1 with maintenance or stimulation of beneficial TNFR2 function represents a promising strategy for suppressing inflammation and maintaining neuroprotection for the treatment of a wide range of CNS diseases.

## Supplementary Information

**Supplementary Figure 1: Brain-specific depletion of TNFR1 and TNFR2 in tissues of nTNFR1KO and nTNFR2KO mice.** Allele-specific DNA PCR analysis was used to assess the tissue specificity of Cre-mediated recombination (deletion) events in different tissues of *LoxP*-flanked (“floxed”) *Tnfrsf1a* (**A**) or *Tnfrsf1b* (**B**) sequences in mice (“defloxed”). Deletion of floxed *Tnfrsf1a* and *Tnfrsf1b* alleles was restricted to brain (cortex, hippocampus and cerebellum) and spinal cord of nTNFR1KO and nTNFR2KO mice, and not TNFR1ff or TNFR2ff control mice, respectively. Control tissues are brain from nTNFR1KO (A; Cre ff), nTNFR2KO (B; Cre ff) and WT B6 (+/+) mice.

**Supplementary Figure 2: Neuronal TNFR2 does not affect CPZ demyelination and remyelination**. (**A**) Semiquantitative scoring of demyelination (loss of LFB staining) in the medial corpus callosum of nTNFR2KO and TNFR2ff control naïve (CPZ0) or CPZ-fed CPZ5 and CPZ6+2 mice. Quantitative representation of (**B**) CNPase immunoreactivity by densitometry, (**C**) Iba1 immunoreactivity by % area covered, and (**D**) numbers of APP immunoreactive spheroids/mm2 tissue in the corpus callosum in serial coronal paraffin sections of brain from nTNFR2KO and TNFR2ff control mice. Results are means of 2 (CPZ0; D, CPZ6+2) or ≥5 mice (for all other time points) from one representative of two independent experiments. Statistical significance after comparisons are shown by two-way ANOVA with Bonferroni’s test. * *p* ≤ 0.05, ** *p* ≤ 0.005, *** *p* ≤ 0.001.

**Supplementary Figure 3: Neuronal IKKβ contributes to resolution of CNS T cell infiltration during CPZ remyelination. (A** and **B**) Differential expression of the myelin markers *Mbp* and *Olig2* relative to *GusB* in total mRNA isolates isolated from nIKKβKO and control IKKβff brains from naïve (CPZ0) or CPZ-fed CPZ2, CPZ3, CPZ5 and CPZ6+1 mice. (**Ci**) CD3 immunostaining of T cells in serial brain coronal paraffin sections from nIKKβKO and control IKKβff mice during CPZ demyelination and remyelination. Scale bars: 100 μM. (**Cii**) Numbers of CD3-immunoreactive T cells/mm^2^ tissue counted in coronal paraffin sections through the corpus callosum of brain from nIKKβKO and control IKKβff mice represented in Ci. (**D**) Quantitative representation of neurofilament H phosphorylated (SMI 31) immunoreactivity in the corpus callosum of nIKKβKO and control IKKβff mice by densitometry. Results are means of 2 (CPZ6+1) or 3-5 mice from one representative of two independent experiments. Statistical significance after comparisons are shown by two-way ANOVA with Bonferroni’s test (A and B) or Student’s t-test (). * *p* ≤ 0.05, ** *p* ≤ 0.005.

**Supplementary Figure 4: Preconditioning of astrocyte-neuron co-cultures with solTNF provides neuroprotection against NMDA excitotoxicity.** (**A)** Images from neuron-astrocyte co-cultures at day *in vitro* 7 (NA-DIV7) stained with Hoechst, anti-NeuN for post-mitotic neurons, anti- GFAP for astrocytes and their overlay. Scale bar: 20 μM. (**B**) Neuron-astrocyte co-cultures (NA-DIV7) were incubated with 100 ng/ml human (h) or mouse (m) TNF for 24 h, excitotoxic death was induced by addition of 50 μΜ NMDA/ 10 μΜ glycine on NA-DIV8 and death was measured after 22 h (NA-DIV9) by Hoechst staining. Results shown are means ± SEM of triplicate samples from one representative of five independent experiments. Statistical significance after pairwise comparisons are shown by Student’s t-test. * *p* ≤ 0.05, ** *p* ≤ 0.005, *** *p* ≤ 0.001.

## Data Availability

The datasets used and/or analyzed during the current study are available from the corresponding author on reasonable request.

## Abbreviations

AIF: Apoptosis inducing factor
APP: Amyloid precursor protein
B6: C57BL/6 mice
*Ccl2*: Mouse C-C motif chemokine ligand 2 gene
CFA: Complete Freund’s adjuvant
CNPase: 2′, 3′-Cyclic nucleotide 3′-phosphodiesterase
CPZ: Cuprizone
CPZn: Weeks of cuprizone feeding
CNS: Central nervous system
Cre: Cre recombinase from bacteriophage P1
*Cxcl16*: Mouse C-X-C motif chemokine ligand 16 gene
DIV: Days in vitro
E: Embryonic day
EAE: Experimental autoimmune encephalomyelitis
floxed: *LoxP*-flanked DNA sequence
GFAP: Glial fibrillary acidic protein
*Gusb*: Mouse glucuronidase beta gene
*H2-Ab1*: Mouse H-2 class II histocompatibility antigen, A beta chain gene
hTNF: Human tumor necrosis factor
Iba1: Ionized calcium-binding adaptor molecule 1
IHC: Immunohistochemistry
IKKβ: Inhibitor of nuclear factor kappa-B kinase subunit beta
IKKβff: Littermate control mice with non-deleted *LoxP*-flanked IKKβ sequence
i.p.: Intraperitoneal
KA: Kainic acid
KI: Knockin
KO: Knockout
LC3B: MAP1-LC3B structural protein of autophagosomal membrane
LFB: Klüver-Barrera Luxol fast blue
*LoxP*: Recognition sequence for Cre from bacteriophage P1
*Mbp*: Mouse myelin basic protein gene
MK801: NMDA receptor antagonist
MOG: Murine myelin oligodendrocyte glycoprotein 35-55 peptide
MS: Multiple sclerosis
NeuN: Neuronal nuclear antigen
NF-κB: Nuclear Factor kappa-light-chain-enhancer of activated B cells
NA-DIV: Neuron-astrocyte co-cultures days in vitro
NMDA: N-methyl-d-aspartate
nTNFR1/2KO: Neuron-specific TNFR1/2 knockout mice
OLG: Oligodendrocyte
*Olig2*: Mouse oligodendrocyte transcription factor 2 gene
OPC: Oligodendrocyte precursor cells
P: Postnatal day
PCR: Polymerase chain reaction
PI3K-PKB/Akt: Phosphatidylinositol 3-kinase and Akt/protein kinase B signaling pathway
PTx: *Bordetella pertussis* toxin
SMI 31: Neurofilament H phosphorylated
*Snap25*: Mouse synaptosomal-associated protein 25kDa gene
solTNF: Soluble TNF
tmTNF: Transmembrane TNF
TNF: Tumor necrosis factor
TNFR1/2ff: Littermate control mice with non-deleted *LoxP*-flanked TNFR1/2 sequences
TNFR1: TNF receptor 1
TNFR2: TNF receptor 2
TNFR2ag: TNFR2 agonist
WT: Wild-type mice
