# Supplementary figures and images for: Fundamentally different roles of neuronal TNF receptors in CNS pathology: TNFR1 and IKKβ promote microglial responses and tissue injury in demyelination while TNFR2 protects against excitotoxicity in mice
Source: J Neuroinflammation. 2021 Sep 26;18:222. doi: 10.1186/s12974-021-02200-4 (PMC8466720; doi:10.1186/s12974-021-02200-4)

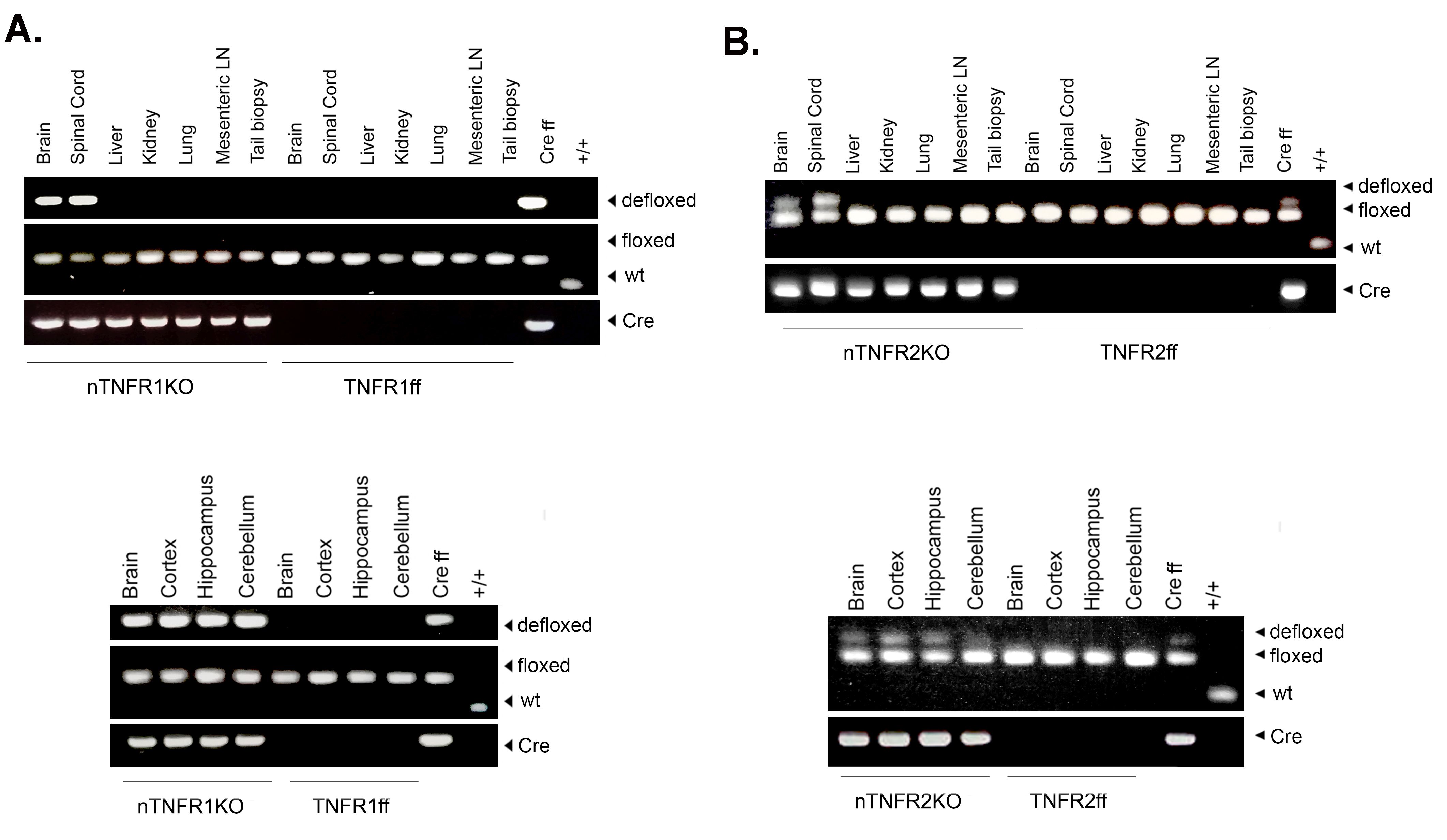

Supplement: Supplementary file 1 — Supplementary Figure 1: Brain-specific depletion of TNFR1 and TNFR2 in tissues of nTNFR1KO and nTNFR2KO mice. Allele-specific DNA PCR analysis was used to assess the tissue specificity of Cre-mediated recombination (deletion) events in different tissues of LoxP-flanked (“floxed”) Tnfrsf1a (A) or Tnfrsf1b (B) sequences in mice (“defloxed”). Deletion of floxed Tnfrsf1a and Tnfrsf1b alleles was restricted to brain (cortex, hippocampus and cerebellum) and spinal cord of nTNFR1KO and nTNFR2KO mice, and not TNFR1ff or TNFR2ff control mice, respectively. Control tissues are brain from nTNFR1KO (A; Cre ff), nTNFR2KO (B; Cre ff) and WT B6 (+/+) mice. [file 12974_2021_2200_MOESM1_ESM.jpg]

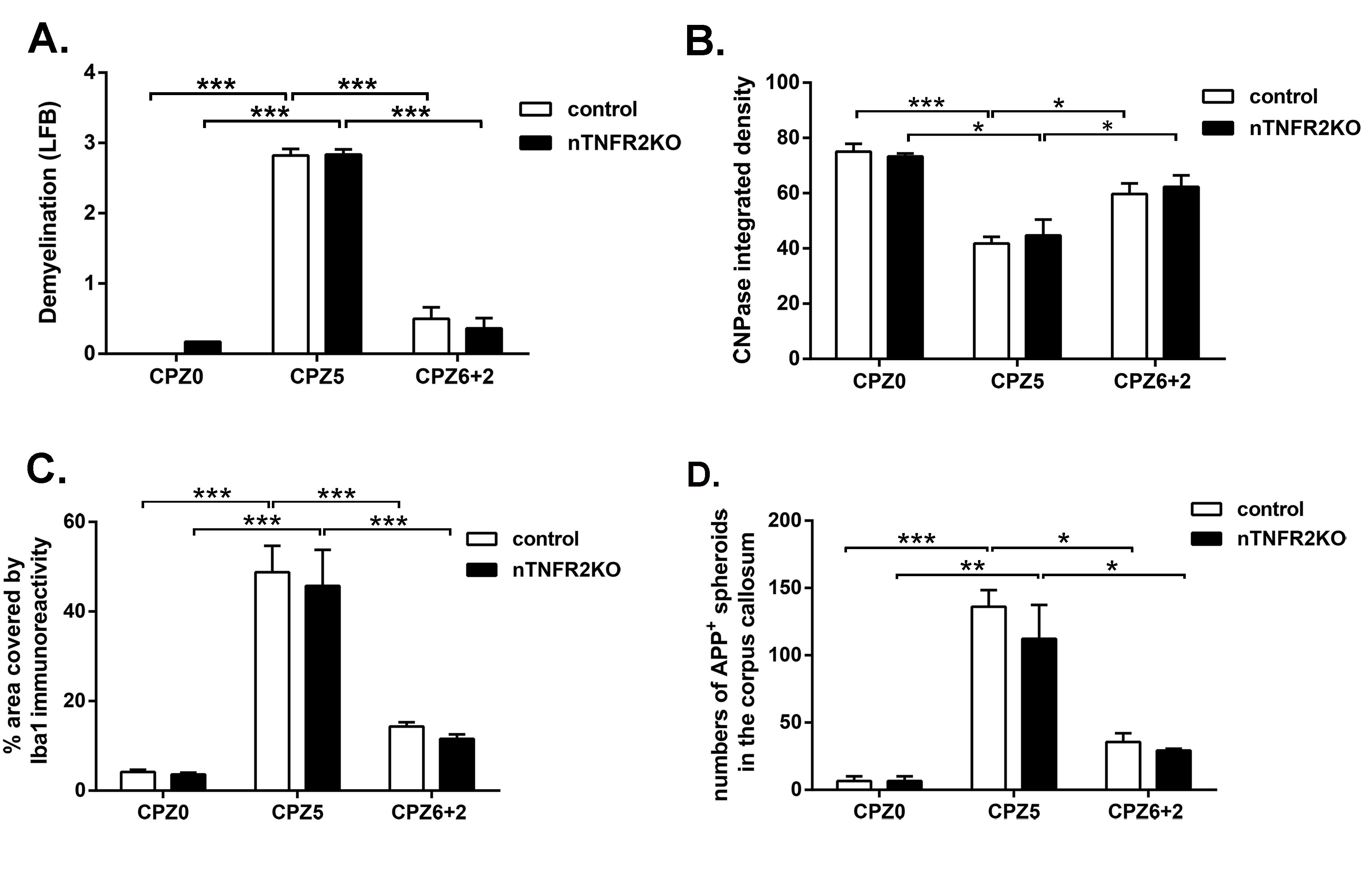

Supplement: Supplementary file 2 — Supplementary Figure 2: Neuronal TNFR2 does not affect CPZ demyelination and remyelination. (A) Semiquantitative scoring of demyelination (loss of LFB staining) in the medial corpus callosum of nTNFR2KO and TNFR2ff control naïve (CPZ0) or CPZ-fed CPZ5 and CPZ6+2 mice. Quantitative representation of (B) CNPase immunoreactivity by densitometry, (C) Iba1 immunoreactivity by % area covered, and (D) numbers of APP immunoreactive spheroids/mm2 tissue in the corpus callosum in serial coronal paraffin sections of brain from nTNFR2KO and TNFR2ff control mice. Results are means of 2 (CPZ0; D, CPZ6+2) or ≥5 mice (for all other time points) from one representative of two independent experiments. Statistical significance after comparisons are shown by two-way ANOVA with Bonferroni’s test. * p ≤ 0.05, ** p ≤ 0.005, *** p ≤ 0.001. [file 12974_2021_2200_MOESM2_ESM.jpg]

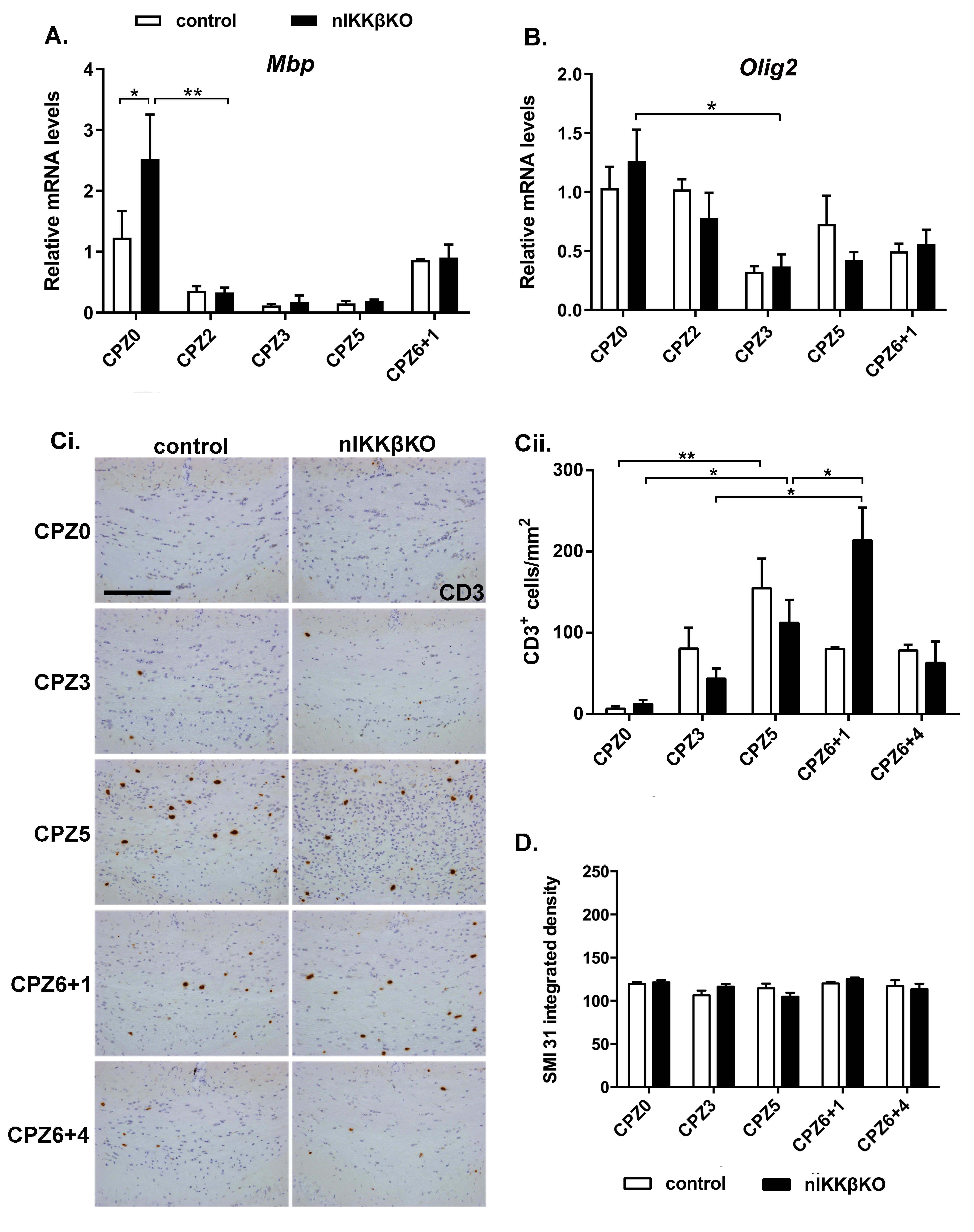

Supplement: Supplementary file 3 — Supplementary Figure 3: Neuronal IKKβ contributes to resolution of CNS T cell infiltration during CPZ remyelination. (A and B) Differential expression of the myelin markers Mbp and Olig2 relative to GusB in total mRNA isolates isolated from nIKKβKO and control IKKβff brains from naïve (CPZ0) or CPZ-fed CPZ2, CPZ3, CPZ5 and CPZ6+1 mice. (Ci) CD3 immunostaining of T cells in serial brain coronal paraffin sections from nIKKβKO and control IKKβff mice during CPZ demyelination and remyelination. Scale bars: 100 μM. (Cii) Numbers of CD3-immunoreactive T cells/mm2 tissue counted in coronal paraffin sections through the corpus callosum of brain from nIKKβKO and control IKKβff mice represented in Ci. (D) Quantitative representation of neurofilament H phosphorylated (SMI 31) immunoreactivity in the corpus callosum of nIKKβKO and control IKKβff mice by densitometry. Results are means of 2 (CPZ6+1) or 3-5 mice from one representative of two independent experiments. Statistical significance after comparisons are shown by two-way ANOVA with Bonferroni’s test (A and B) or Student’s t-test (). * p ≤ 0.05, ** p ≤ 0.005. [file 12974_2021_2200_MOESM3_ESM.jpg]

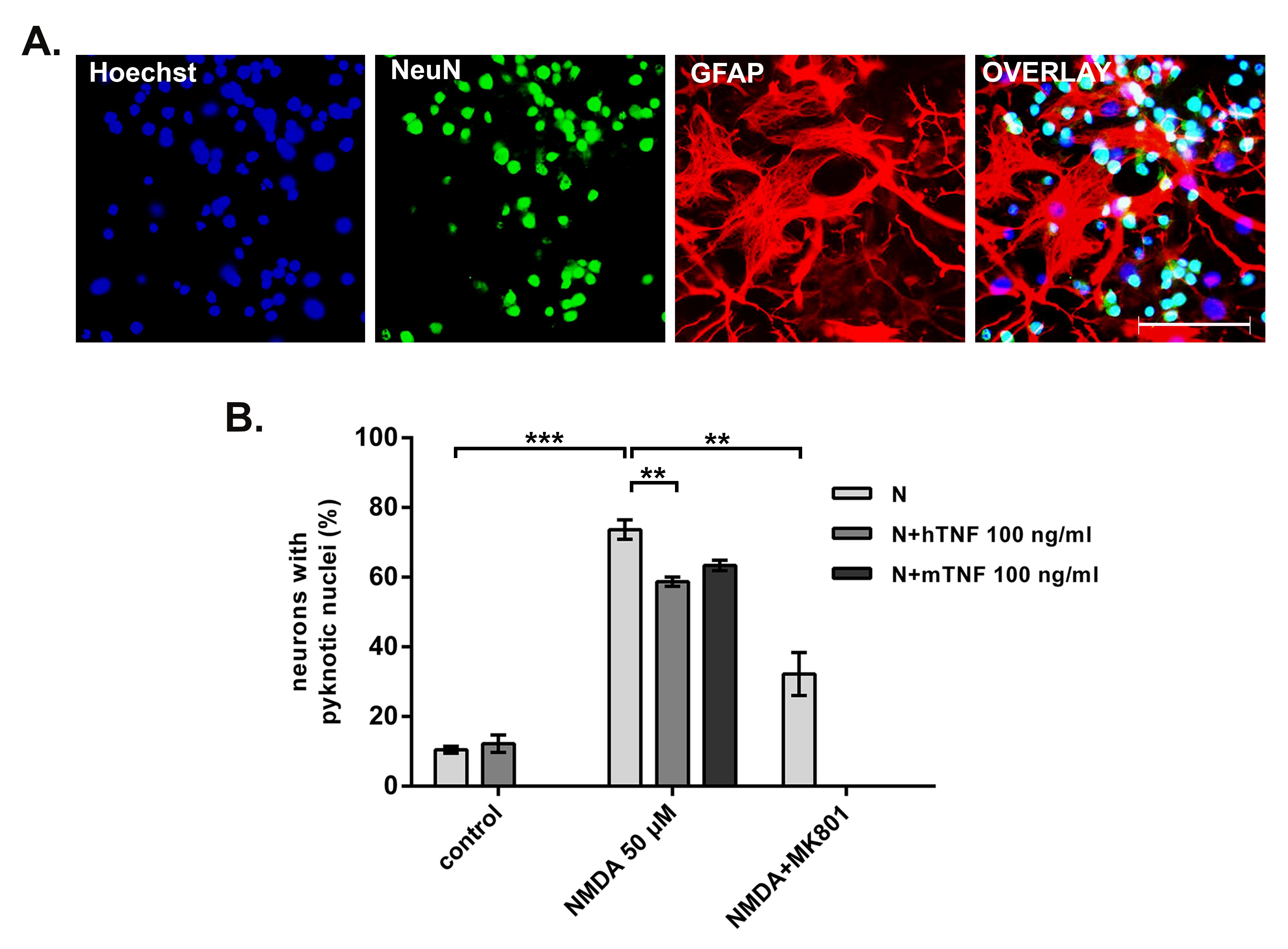

Supplement: Supplementary file 4 — Supplementary Figure 4: Preconditioning of astrocyte-neuron co-cultures with solTNF provides neuroprotection against NMDA excitotoxicity. (A) Images from neuron-astrocyte co-cultures at day in vitro 7 (NA-DIV7) stained with Hoechst, anti-NeuN for post-mitotic neurons, anti- GFAP for astrocytes and their overlay. Scale bar: 20 μM. (B) Neuron-astrocyte co-cultures (NA-DIV7) were incubated with 100 ng/ml human (h) or mouse (m) TNF for 24 h, excitotoxic death was induced by addition of 50 μΜ NMDA/ 10 μΜ glycine on NA-DIV8 and death was measured after 22 h (NA-DIV9) by Hoechst staining. Results shown are means ± SEM of triplicate samples from one representative of five independent experiments. Statistical significance after pairwise comparisons are shown by Student’s t-test. * p ≤ 0.05, ** p ≤ 0.005, *** p ≤ 0.001. [file 12974_2021_2200_MOESM4_ESM.jpg]
